# Identification of tumor-agnostic biomarkers for predicting prostate cancer progression and biochemical recurrence

**DOI:** 10.3389/fonc.2023.1280943

**Published:** 2023-10-26

**Authors:** William Lautert-Dutra, Camila M. Melo, Luiz P. Chaves, Francisco C. Souza, Cheryl Crozier, Adam E. Sundby, Elizabeth Woroszchuk, Fabiano P. Saggioro, Filipe S. Avante, Rodolfo B. dos Reis, Jeremy A. Squire, Jane Bayani

**Affiliations:** ^1^ Department of Genetics, Medical School of Ribeirao Preto, University of Sao Paulo, Ribeirao Preto, Brazil; ^2^ Division of Urology, Department of Surgery and Anatomy, Medical School of Ribeirao Preto, University of Sao Paulo, Ribeirao Preto, Brazil; ^3^ Diagnostic Development, Ontario Institute for Cancer Research, Toronto, ON, Canada; ^4^ Department of Pathology, Ribeirao Preto Medical School, University of Sao Paulo, Ribeirao Preto, Brazil; ^5^ Department of Pathology and Molecular Medicine, Queen’s University, Kingston, ON, Canada; ^6^ Laboratory Medicine and Pathology, University of Toronto, Toronto, ON, Canada

**Keywords:** gene signature, RNA biomarkers, NanoString BC360, biochemical recurrence, CAPRA-S, TCGA-PRAD

## Abstract

The diverse clinical outcomes of prostate cancer have led to the development of gene signature assays predicting disease progression. Improved prostate cancer progression biomarkers are needed as current RNA biomarker tests have varying success for intermediate prostate cancer. Interest grows in universal gene signatures for invasive carcinoma progression. Early breast and prostate cancers share characteristics, including hormone dependence and BRCA1/2 mutations. Given the similarities in the pathobiology of breast and prostate cancer, we utilized the NanoString BC360 panel, comprising the validated PAM50 classifier and pathway-specific signatures associated with general tumor progression as well as breast cancer-specific classifiers. This retrospective cohort of primary prostate cancers (*n*=53) was stratified according to biochemical recurrence (BCR) status and the CAPRA-S to identify genes related to high-risk disease. Two public cohort (TCGA-PRAD and GSE54460) were used to validate the results. Expression profiling of our cohort uncovered associations between *PIP* and *INHBA* with BCR and high CAPRA-S score, as well as associations between *VCAN*, *SFRP2*, and *THBS4* and BCR. Despite low levels of the *ESR1* gene compared to *AR*, we found strong expression of the ER signaling signature, suggesting that BCR may be driven by ER-mediated pathways. Kaplan-Meier and univariate Cox proportional hazards regression analysis indicated the expression of *ESR1*, *PGR*, *VCAN*, and *SFRP2* could predict the occurrence of relapse events. This is in keeping with the pathways represented by these genes which contribute to angiogenesis and the epithelial-mesenchymal transition. It is likely that *VCAN* works by activating the stroma and remodeling the tumor microenvironment. Additionally, *SFRP2* overexpression has been associated with increased tumor size and reduced survival rates in breast cancer and among prostate cancer patients who experienced BCR. *ESR1* influences disease progression by activating stroma, stimulating stem/progenitor prostate cancer, and inducing TGF-β. Estrogen signaling may therefore serve as a surrogate to AR signaling during progression and in hormone-refractory disease, particularly in prostate cancer patients with stromal-rich tumors. Collectively, the use of agnostic biomarkers developed for breast cancer stratification has facilitated a precise clinical classification of patients undergoing radical prostatectomy and highlighted the therapeutic potential of targeting estrogen signaling in prostate cancer.

## Introduction

Prostate cancer (PCa) is the second most common and the fourth most frequent cause of male deaths (1). Most cases are diagnosed with a localized, slowly progressive, and indolent disease. Unfortunately, 20-30% of patients with the localized disease will eventually progress and develop metastasis, with a reduced 5-year survival rate (2).

The histologic and molecular characteristics of PCa are used to manage patient care and guide therapeutic decision-making in this clinically heterogeneous disease (2–5). Gleason-score and tumor grade are used to classify patient tissue biopsies from different areas of the prostate to predict tumor behavior. At the molecular level, androgen receptor-positive PCa and advanced diseases with mutations in DNA damage response genes (e.g., *BRCA1/2*) are eligible for systemic anti-androgen therapy to block the activation of androgen receptors and for treatment with poly (ADP-ribose) polymerase inhibitors (PARPi), respectively (6). However, there are currently insufficient biomarkers to reliably distinguish between an indolent and aggressive disease course at the time of radical prostatectomy (7) or biopsy. As a result, many PCa patients undergo surgery or other treatments resulting in side effects that can considerably impact their quality of life (8).

The development of precision medicine in PCa has involved evaluating various combinations of expression-based prognostic biomarkers (9). Prognostic signatures are often derived by comparing gene expression differences between aggressive and indolent diseases (10–12). Some of the commercial PCa biomarker signatures have focused on specific biological features of progression. For example, the Prolaris test analyzes expression differences of genes known to be involved in cell cycle control (13). The Decipher test has been similarly validated and is comprised of genes related to proliferation, immune function, and cell adhesion (14). Other PCa gene panels have addressed androgen signaling (15) and stem cell functions (16).

Tumor-agnostic biomarkers are molecular signatures or biomarkers used to select therapies regardless of the tumor site of origin. The development of inter-tumor tissue-agnostic biomarkers has revolutionized cancer care (17, 18). For example, the discovery of similar molecular features, such as tumor-mutation burden (TMB), and mismatch repair (MMR) defective tumors, led to the approval of immune checkpoint blockers for carcinomas regardless of their anatomic location (19–22). Breast (BCa) and PCa are the two most common invasive carcinomas in women and men (23). For both organs, steroid hormones, such as estrogen, progesterone, and androgen, are essential for normal development (24, 25). These endocrine-driven tumors have similar lifetime sporadic risks and are subject to germline and somatic mutations (e.g., *BRCA1/2*) (2, 25). Both share common biological features of hormone-dependent development, and hormonal therapies are the main approach to disease control (23, 26), with resistance to primary hormonal treatment as the leading mechanism for disease progression.

Pivotal to the continued understanding of breast pathogenesis and the recognition of the impact of molecular heterogeneity, the seminal work by Perou et al. (27) resulted in the PAM50 classifier. The molecular subtypes luminal A, luminal B, HER2 enriched, and basal-like subtypes have been shown to be prognostic with general concordance to the immunohistochemistry (IHC)-based diagnostic stratification of BCas by ER, PR, and HER2 (28). The classifier, with its resulting risk of recurrence score (ROR), has been commercially available as the Prosigna Breast Cancer Assay (Veracyte Inc.), for prognosis in hormone receptor-positive, HER2-negative early BCa using the NanoString nCounter System (NanoString Technologies) (28). Using PAM50, comparative studies in PCa have shown that they could be similarly classified as luminal or non-luminal (i.e., basal) (29), further demonstrating the similarities in pathobiology. Since RNA gene signature biomarker panels are well-developed in BCa (30), we investigated whether genes or pathway-derived signatures might be common to progression in both tumor types. Improved clinical stratification of PCa patients at the time of first surgery can help to avoid unnecessary follow-up treatments for patients with a more favorable predicted disease course and benefit high-risk patients for rapid precision medicine treatment when eligible. In this way, we explored the use of a curated gene panel related to key molecular pathways included in NanoString’s Breast Cancer 360 (BC360) assay of 776 genes to identify inter-tumor agnostic markers common to breast and prostate cancer; that may be promising for identifying actionable biological targets and pathways that can be putatively repurposed in PCa. These findings will help identify cancers that are more likely to progress to other tissues and provide more novel biomarkers for more precise patient classification at the time of surgery.

## Material and methods

### Tumor cohort

All 53 samples included in the Faculty of Medicine of Ribeirao Preto (FMRP) cohort were primary PCa collected by radical prostatectomy following National Comprehensive Cancer Network (NCCN) clinical practice guidelines (31) in the Department of Surgery and Anatomy, Urology Division at Ribeirao Preto Medical School, Brazil, between 2007 and 2015 (**Table S1**). According to the American College of Pathology guidelines, the smaller prostates were submitted in their entirety. For partial sampling in the setting of larger glands, we followed the protocol of consistently submitting the whole grossly visible tumor (when identified), the tumor and associated periprostatic tissue and margins, along with the entire apical and bladder neck margins and the junction of each seminal vesicle with prostate proper. If there was no grossly visible tumor, a systematic sampling strategy was used that included submitting the posterior aspect of each transverse slice along with a mid-anterior block from each side, and the entire apical and bladder neck margins and the junction of each seminal vesicle with the prostate. The patients were classified according to the presence of biochemical recurrence (BCR), defined as PSA>0.2 ng/ml within six months after radical prostatectomy. We also estimated the risk of prognostic PCa recurrence after fist-line surgery using the Cancer of The Prostate Risk Assessment Score (CAPRA-S) (32). This score estimator is based on clinical and pathological information available before and after radical prostatectomy, and predicts the relative risk of biochemical progression. Patients were evaluated based on variables including pre-treatment PSA level, pathological Gleason Score, surgical margin, extracapsular extension, seminal vesicle invasion, and lymph node invasion, contributing to their CAPRA-S score, ranging from 1 to 12 (32). Patients with low CAPRA-S scores were those with values between 0-2, those with intermediate scores had CAPRA-S scores ranging from 3-5, and those with high scores had CAPRA-S scores between 6-12. Patient outcome data were collected to the last follow-up date. This retrospective study was approved by the Ethics Committee in Research of Hospital of Ribeirão Preto, São Paulo, Brazil (HCRP) number CAAE 60032122.8.0000.5440 and the Ethics Board of the University of Toronto (Protocol: 00043323).

### RNA isolation

RNA was isolated from tissues with tumor-rich areas previously marked by a pathologist (FPS) which represent the highest Gleason pattern. Sections were processed at the Ontario Institute for Cancer Research, Toronto, Canada (OICR) using a dual DNA and RNA extraction as previously described (33, 34). Briefly, hematoxylin and eosin slides were prepared for all the formalin-fixed paraffin-embedded (FFPE) tissues. Histologic analysis of all the slides was performed in the pathology department, and all tumor areas were carefully marked by an experienced pathologist (FPS). Within each marked tumor-rich area, the percentage of tumor cells (range 70-95% tumor-rich) was estimated and recorded (**Table S2**). Adjacent slides for each tumor were prepared, and the same areas of interest were macrodissected for RNA extraction.

### Transcription analysis

The Human nCounter Breast Cancer 360 (NanoString Technologies Inc., Seattle, WA, USA) is a 776 gene panel designed to characterize breast cancer-specific gene expression patterns associated with breast cancer tumor progression. The panel contains several proprietary signatures generated by NanoString, describing key aspects of breast cancer biology and immune oncology to aid tumor classification. Among these signatures is the PAM50 Signature (28), which classifies tumors into one of four molecular subtypes (Luminal A, Luminal B, HER2-Enriched, and Basal-like) associated with tumor biology and patient prognosis as well as a Genomic Risk Score. The panel also includes the Tumor Inflammation Signature (35), which measures the tumor’s pre-existing, peripherally suppressed adaptive immune responses. A description of the NanoString proprietary signatures and the genes comprising these signatures is found in **Supplementary Table S3**. The generation of scores for these proprietary signatures was performed by NanoString.

For prognostic biomarker discovery, raw expression data from the BC360 was loaded onto the *nSolver* software v4.0 (NanoString Technologies) to perform the quality control (QC analysis) and to build the transcript matrix for downstream analysis (**Figures S1A, B**). For differential expression, we used *DESeq2* v1.34.0 with BCR and risk classification as the design factors (36). We performed over-representation enrichment analysis (ORA) in the differential expressed genes using the *clusterProfiler* v4.0 (37). Because PCa is a hormonally driven cancer, we dichotomized expression levels of *AR*, *PGR*, and *ESR1* based on mean gene expression for each gene; we then used this categorical data to generate the final classification regarding the patient’s gene expression for *AR*, *PGR*, and *ESR1*.

### Statistical analysis

The data processing and downstream analysis were completed in *RStudio* software (R Foundation for Statistical Computing, *R* v4.1.2 “Bird Hippie”). All results were plotted using *ggplot2*, *pheatmap*, or Broad Institute’s *Morpheus* software (38). For the validation cohort, we used RNA-seq data from the prostate adenocarcinoma cohort from The Cancer Genome Atlas (PRAD-TCGA, *n* = 420) (39) and from GSE54460 (*n* = 106) (40). Only patients with matched BCR data were used in the study. To replicate the potential features of the BC360 panel, we further subset the expression matrix of the validation cohorts to contain just the intersection of genes in the BC360 panel, yielding a matrix totaling 758 common genes (41). Genes were considered differentially expressed when log2 foldchange > 0.5 for the BC360 panel, and a more rigorous threshold of > 0.58 was used for the TCGA-PRAD and GSE54460. Adjusted p-value controlling for multiple testing was performed and false-discovery rate (FDR) < 0.05 was reported. For the enrichment analysis, we used a cut-off value of 0.05 to consider the ORA of Hallmarks’ terms. Further, the dichotomized expression based on mean gene expression levels of *AR*, *PGR*, *ESR1*, and the DEGs associated with BCR and CAPRA-S for each gene was used to estimate the time-to-detection of BCR. The log-rank test was used to estimate the time-to-detection of BCR in Kaplan Meier curves (BCR free-survival, BCRFS), and the Cox proportional hazard (PH) regression model was computed using the *survival* R package v3.4.0 (**Figure S2**).

## Results

### Molecular subtyping and propriety signature findings using the BC360 assay

**Figure 1:**
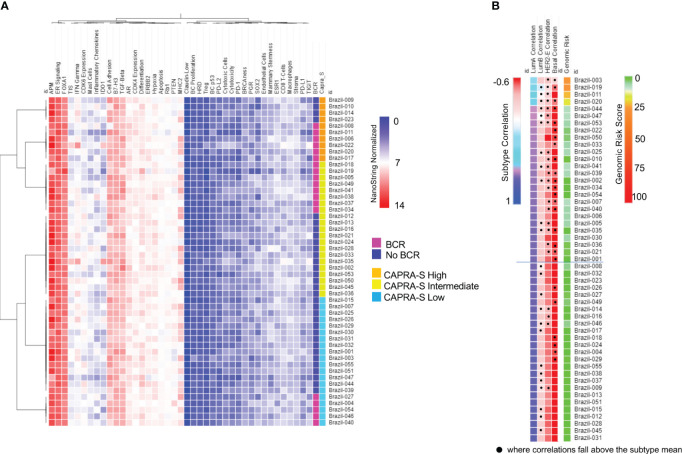
NanoString BC360 Signatures. **(A)** Unsupervised heatmap of BC360 signatures pathway-specific or prognostic signatures stratified by CAPRA-S score and BCR status. In this panel, there is a strong expression of the ER Signaling Signature, with comparably intermediated and low expression of AR and PGR, respectively. **(B)** PAM50 classifier with all 53 cases was identified as Luminal **(A)** Heatmaps were generated using Broad Institute’s Morpheus software https://software.broadinstitute.org/morpheus/andpheatmap.

BC360 analysis was performed using NanoString’s proprietary workflow (NanoString Technologies) (**Figure S2**) to generate pathway-specific or prognostic signatures (**Figure 1**; **Table S3**). Using the signature results and individual prognostic genes which were normalized and scaled, the cohort generally showed similar features including relatively low expression of a homologous recombination deficiency (HRD) signature and markers typically associated with immune “cold” tumors, including PD-L-1, PD1, and PD-L2 (**Figure 1A**). Conversely, higher expression was seen consistently across the cohort for the ER Signaling Signature, despite a relatively overall lower expression of *ESR1* and *PGR* as individual genes. In contrast, comparatively higher and variable expression of AR was seen, as expected. Variable expression of the Tumor Inflammation Signature (TIS) was observed suggesting differences between the cases in the abundance of peripherally suppressed adaptive immune response genes. When clustered according to CAPRA-S and BCR status, the signature expressions organized patients into two main groups that were differentiated by the variable expression of genes or gene signatures associated with immune response genes including: the IDO1 and genes associated with inflammatory cytokines and the microenvironment; IFNγ signaling; and the TIS signature. Differential expression of ERBB2, CDK4, PTEN, and genes comprising the signatures of MHC2 and cellular differentiation and the epithelial to mesenchymal transition (EMT) were also noted. Of the two main groupings, one was comprised of CAPRA-S high and intermediate cases, which included many patients who experience BCR. This contrasted with the large group of patients of CAPRA-S low and intermediate cases, for which many did not experience BCR.

**Figure 2:**
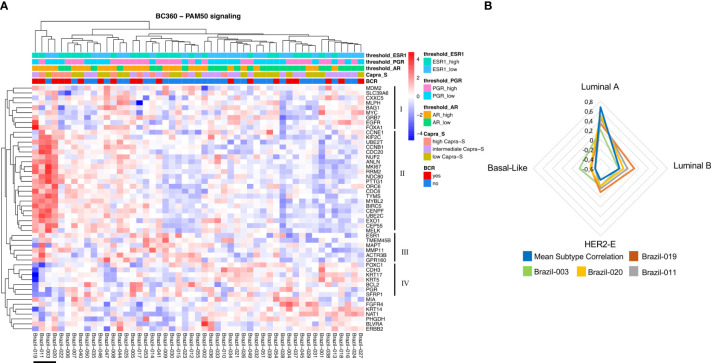
NanoString PAM50 and breast cancer subtypes. **(A)** Unsupervised heatmap of 50 genes encompassing the PAM50 signature in the Nanostring BC360 panel. A clear clustering for patients associated with the other subtypes (Brazil-019, -011, -003, and -020) can be observed on the left side of the panel. This group of patients also shows two major clusters with opposite expression profiles (II and III). For each patient, the level of expression [divided by quartiles (see Methods)] is displayed for the androgen receptor (*AR*), *PGR*, and estrogen receptor 1 (*ESR1*). **(B)** Gene expression features for patients associated with the other subtypes. For this group of patients, the mean subtype correlation was higher and shifted towards the Luminal B, HER2-E, and Basal-like subtypes when compared with other samples. Mean correlation Luminal A: 0.677; Luminal B: -0.201; HER2-E: -0.364; Basal-like: -0.49. The signature scores were used to perform hierarchical clustering for both patients and gene signatures in the context of CAPRA-S and BCR. Heatmaps were generated using Broad Institute’s Morpheus software https://software.broadinstitute.org/morpheus/andpheatmap.

All 53 cases from the FMRP cohort were identified as Luminal A using the validated PAM50 classifier (**Figure 1B**), although we observed shifts towards correlations to the Luminal B, HER2E, and Basal-like subtypes when the raw correlation coefficients, were analyzed (**Figures 1B**, **2**). Furthermore, the Nanostring assay was able to correctly classify a control (basal subtype) among all cases (**Table S4**). Utilizing the mean Luminal A correlation coefficient as a cut-off (0.67), we demonstrated that some cases possessed gene expression features associated with the other subtypes. Indeed, in those patients: Brazil-003, -011, -019, and -020, there was an observed positive shift from the mean correlation towards more Luminal B (mean correlation=-0.20), HER2E (mean correlation=-0.36), and Basal-like (mean correlation=-0.49) subtypes, with the concomitant negative shift away from the Luminal A correlation mean, though not sufficiently significant to be classified as such. The Genomic Risk Score, used to evaluate the relative risk of recurrence in BCa, was shown to increase amongst these PCas, reflecting a more aggressive cancer phenotype as it becomes less Luminal A-like (**Figure 1B**). In the cases of Brazil-003, 011, -019, and -020, the Genomic Risk Scores were 25, 28, 22, and 18 respectively, significantly higher than the average Genomic Risk Score of 4.2 across the FMRP cohort. Unsupervised clustering of the 50 genes of the PAM50 signature (**Figure 2**) clearly demonstrated these four cases as possessing similar features with comparatively higher expression of proliferation and cell cycle genes (e.g., *MYC*, *EGFR*, *FOXA1*, *ORC6*, and *MELK*) and receptor tyrosine kinases (*EGFR*); and observed comparatively lower expression of *FOXC1*, *CDH3*, and *KRT17*, among others. Correlations to clinical BCR and CAPRA-S show altered genes related to the EMT.

**Figure 3:**
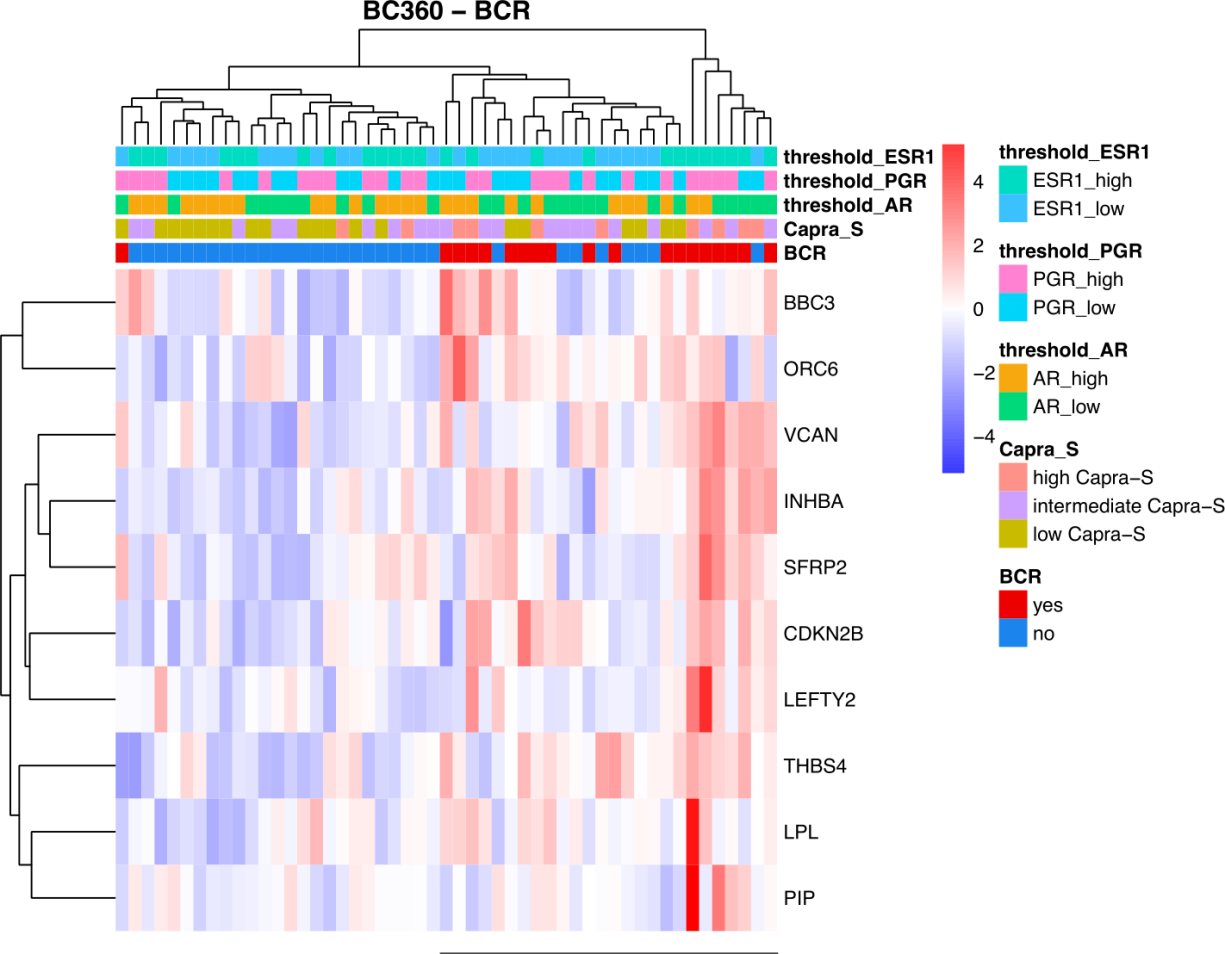
Summary of the retrospective FMRP cohort using the NanoString BC360 Unsupervised heatmap of the DEGs based on biochemical recurrence (BCR). There is an observed clustering of BCR patients and association with higher expression of *ER1* and *PGR* (horizontal bar). For each patient, the level of expression (divided by quartiles (see Methods)) is displayed for the androgen receptor (*AR*), progesterone receptor (*PGR*), and estrogen receptor 1 (*ESR1*). Patients that remained disease-free after radical prostatectomy were used as the reference. (Log2FC > 0.5, P-adjusted < 0.05 (FDR)).

**Figure 4:**
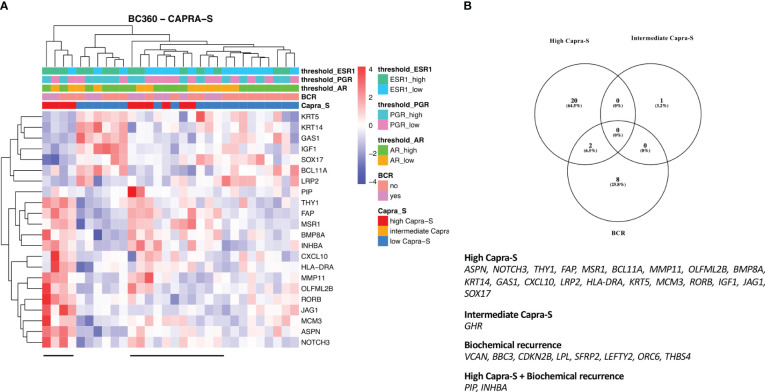
Summary results of FMRP cohort based on the CAPRA-S using the NanoString BC360 panel - Unsupervised heatmap of the DEGs comparing **(A)** “High” risk patients classified by CAPRA-S. “High” risk patients showed clustering (horizontal bar). **(B)** Venn diagram shows the intersection of the DEGs from the NanoString BC360 panel analysis according to BCR and CAPRA-S status. For each patient, the level of expression (divided by quartiles (see Methods)) is displayed for the androgen receptor (AR), progesterone receptor (PGR), and estrogen receptor 1 (ESR1). Log2FC > 0.5, P-adjusted < 0.05 (FDR).

To leverage the full potential of this 776-gene panel, we examined the differentially expressed genes (DEGs) (log2 foldchange > 0.5, padj (FDR) < 0.05), comparing tumors from patients with BCR to those who remained disease-free in addition to CAPRA-S. Through this comparison, we identified significant up-regulation of *VCAN*, *BBC3*, *CDKN2B*, *LPL*, *SFRP2*, *THBS4, INHBA*, *PIP*, *LEFTY2*, and *ORC6* genes in BCR patients (**Table 1**). Unsupervised analysis of these 10 upregulated genes showed apparent clustering of the patient group enriched for those who experienced a BCR (**Figure 3**). We then analyzed the transcriptome of patients based on the CAPRA-S intermediate and high relative risk of biochemical progression (**Table 2**). In the unsupervised analysis, the “High” risk patients predominantly clustered together (**Figure 4A**) when compared to the “Low” risk group. Amongst “Intermediate” risk samples, there was and observed upregulation of one gene, *GHR*. Using the results obtained from the BCR and CAPRA-S comparison, a list of DEGs related exclusively to each clinical phenotype and a set of DEGs common to all conditions was generated (**Figure 4B**). We found 8 DEGs exclusively associated with BCR (*VCAN*, *BBC3*, *CDKN2B*, *LPL, SFRP2, LEFTY2, ORC6*, and *THBS4*); 20 DEGs were exclusively found through CAPRA-S. Additionally, when comparing both BCR and CAPRA-S, we identified two DEGs - *PIP*, *INHBA*) (as shown in **Figure 4B**).

**Table 1:** Transcriptome change associated with BCR in the FMRP cohort.

	Gene	Log2FC	padj	Protein	Role in progression and biology of PCa	Citations
**BCR**	*VCAN*	0.671	0.006	Versican	VCAN expression has important roles in the tumor microenvironment, immune cell infiltration and extracellular matrix remodeling	(42)
	*BBC3*	0.448	0.01	BCL2 binding component 3	BBC3 is a critical mediator of apoptosis in response to apoptotic stimuli	(43)
	*CDKN2B*	0.518	0.01	cyclin dependent kinase inhibitor 2B	Functions as a cell growth regulator that controls cell cycle G1 progression	(44)
	*INHBA*	0.706	0.01	inhibin beta A subunit	*INHBA* (Activin A) activates NF-κB and is associated with higher Gleason score PCa	(45, 46)
	*LPL*	0.883	0.01	lipoprotein lipase	hydrolyzes triacylglycerols and phospholipids from lipoproteins in extracellular compartment	(47)
	*SFRP2*	0.636	0.01	secreted frizzled related protein 2	*SFRP2* affects TME by regulating Wnt signaling and influencing tumor angiogenesis	(48, 49)
	*PIP*	4.891	0.01	prolactin induced protein	Function in human reproductive and immunological system	(50)
	*LEFTY2*	0.709	0.01	left-right determination factor 2	Member of the TGF-β superfamily and serves as a repressor of TGF-β signaling	(51)
	*ORC6*	0.421	0.03	origin recognition complex subunit 6	crucial for the initiation of DNA replication and cell cycle progression until the late mitosis phase	(52)
	*THBS4*	0.736	0.03	thrombospondin 4	*THBS4* affects cancer stem cell-like properties in PCa by its regulation of the PI3K/Akt pathway.	(53, 54)

List of DEGs derived from the BC360 panel according to BCR status. The analysis used no BCR as the reference. The genes were considered DE when Log2FC > 0.5, P-adjusted < 0.05 (FDR).

**Table 2:** Transcriptome change associated with CAPRA-S in the FMRP cohort.

	Gene	Log2FC	padj	Protein	Role in progression and biology of PCa	Citations
**CAPRA-S**	*ASPN*	1.45	<0.01	asporin	Expression of ASPN is correlated with decreased time to biochemical recurrence and reactive stroma	(54)
	*NOTCH3*	0.673	<0.01	notch 3	*NOTCH1-4* expression was associated with disease progression, prognosis, and immune cell infiltration.	(55)
	*PIP*	6.092	<0.01	prolactin induced protein	Function in human reproductive and immunological system	(50)
	*THY1*	0.894	<0.01	Thy-1 cell surface antigen	*THY1* over-expressed in PCa-associated fibroblasts.	(56)
	*FAP*	1.028	<0.01	fibroblast activation protein alpha	FAP overexpression are linked to CAF, tumor invasion, lymph node metastasis, and decreased overall survival	(57, 58)
	*MSR1*	0.794	<0.01	macrophage scavenger receptor 1	Helpful as an additional diagnostic biomarker for PCa.	(59)
	*BCL11A*	-0.87	0.002	B-cell CLL/lymphoma 11A	BCL11A knockdown suppresses prostate cancer cell lines proliferation and invasion	(60)
	*MMP11*	1.001	0.002	matrix metallopeptidase 11		(61)
	*OLFML2B*	0.914	0.006	olfactomedin like 2B		
	*BMP8A*	0.709	0.01	bone morphogenetic protein 8a	*BMP8A* increased expression associated with early BCR.	(62)
	*KRT14*	-1.297	0.01	keratin 14	High expression being significantly correlated with poor differentiation in Gleason grading, pathologic tumor stage 4 (pT4), and positive-bone metastasis (p<0.05)	(61)
	*GAS1*	-0.662	0.02	growth arrest specific 1	*GAS1RR* (an immune-related enhancer RNA) represses *GAS1*, associated with BR-free survival in PCa.	(63)
	*CXCL10*	1.172	0.02	C-X-C motif chemokine ligand 10	CXCL10 coexpression with *CXCR3* predictor of metastatic recurrence.	(64)
	*INHBA*	0.799	0.02	inhibin beta A subunit	*INHBA* (Activin A) activates NF-κB and is associated with higher Gleason score PCa	(45, 46)
	*LRP2*	-0.623	0.02	LDL receptor related protein 2	Endocytic receptor that internalizes testosterone bound to sex hormone-binding globulin into prostate cells	(65)
	*HLA-DRA*	0.632	0.03	major histocompatibility complex, class II, DR alpha	Antigen presentation in TME	
	*KRT5*	-0.89	0.03	keratin 5	Loss of keratin 5 expression is closely associated with acquisition of a tumorigenic phenotype by rat bladder non-tumorigenic cells.	(66)
	*MCM3*	0.311	0.03	Minichromosome maintenance complex component 3	MCM3 is upregulated in mesenchymal phenotype of human prostate cancer cells and advanced human prostate cancer specimens	(67)
	*RORB*	0.681	0.04	RAR related orphan receptor B	*RORB* correlated with age, tumor status, lymph node status, disease-free survival (DFS), progression-free survival (PFS), and overall survival (OS)	(68)
	*IGF1*	-0.649	0.04	insulin like growth factor 1	Increased insulin-like growth factor 1 was associated with increased risk of prostate cancer.	(69)
	*JAG1*	0.553	0.04	jagged 1	*JAG1* upregulation results in increased inflammatory foci in TME of tumors in *Pten*-deficient mice.	(70)
	*SOX17*	-0.543	0.04	SRY-box 17	*SOX17* and Notch’s axis associated with enzalutamide resistance in CRPC models.	(71)

List of DEGs derived from the BC360 panel accordingly to CAPRA-S classification. The genes were considered DE when Log2FC > 0.5, P-adjusted < 0.05 (FDR).

When analysing patients according to BCR status in the validation cohort (TCGA-PRAD), we confirmed 4 DEGs also identified in our discovery cohort (*ORC6*, *PIP*, *LEFTY2*, and *INHBA*) (**Figure S5**). Furthermore, using the CAPRA-S “Intermediate” and “High” classifications, we confirmed differential expression of 5 and 14 DEGs also identified in the BC360 cohort (**Figure S3**). Notably, we also detected the differential expression of *INHBA* common in both BCR and CAPRA-S classification systems (**Figure S3**). The GSE54460 cohort showed no DEGs in common with our FMRP discovery cohort when we compared BCR or CAPRA-S groups.

The relative expression of the *AR*, *ESR1*, *ESR2*, and *PGR* in the BCR-positive group to patients without BCR was compared to investigate any possible relationships between the steroid receptors driving the transcriptomic alterations. In our retrospective FMRP cohort, none of the studied receptors showed significant alteration in expression (**Figure S4A**). In contrast, the TCGA-PRAD-BC360 showed a high expression of *AR* (*p* < 0.05) and a lower expression of *PGR* (*p* < 0.05) among the BCR patients (**Figure S4B**).

### BCR and CAPRA-S risk score show different expressions of genes in pathways involved in tumor progression and tumor microenvironment activation

To further understand the transcriptional alterations driving BCR in our cohort, we use enrichment analysis to investigate the functional activity of DEGs. The DEGs in the BCR group were associated with the TGF-β signaling pathway, with the involvement of *CDKN2B*, *INHBA*, and *LEFTY2* (**Table 3**). Interestingly, the relative expression of the normalized gene count of *TGFβ1* shows an increased expression pattern for the BCR patients (**Figure S5**). The DEGs derived from the transcriptome comparison using the CAPRA-S classification also showed significant involvement in several hallmark cancer pathways. These genes were involved in EMT, Notch signaling, TNF-alpha signaling, Allograft Rejection, KRAS signaling, and Inflammatory response. Further enrichment of the DEGs from our validation cohorts also confirmed the involvement of some pathways uncovered in our retrospective FMRP cohort (**Tables S5**–**S7**).

**Table 3:** Enrichment analysis for DEGs associated with BRC and CAPRA-S.

	Term	Adjusted P-value	Genes
BCR	Angiogenesis	0.002	VCAN; LPL
	Epithelial Mesenchymal Transition	0.03	VCAN; INHBA
CAPRA-S	Epithelial Mesenchymal Transition	<0.01	FAP; GAS1; THY1; INHBA
	Notch Signaling	<0.01	NOTCH3; JAG1
	Apical Surface	<0.01	GAS1; THY1
	TNF-alpha Signaling *via* NF-kB	<0.01	CXCL10; JAG1; INHBA
	Allograft Rejection	<0.01	HLA-DRA; THY1; INHBA
	KRAS Signaling Up	<0.01	MMP11; CXCL10; INHBA
	Inflammatory Response	<0.01	MSR1; CXCL10; INHBA

List of ORA enriched terms according to MSigDB cancer Hallmarks terms. The DEGs used for enrichment were filtered with Log2FC > 0.5, P-adjusted < 0.05 (FDR).

### ESR1, PGR, VCAN, and SFRP2 high expression are associated with early relapse

**Figure 5:**
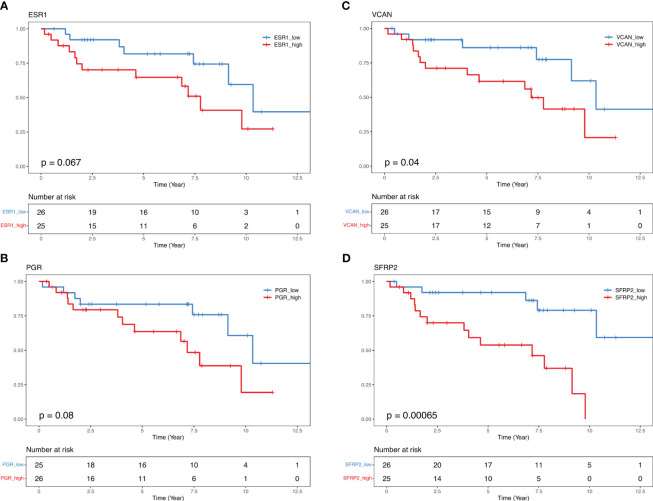
Kaplan-Maier curves illustrate biochemical recurrence free-survival for *ESR1*, *PGR*, *VCAN*, and *SFRP2*. Median 50% progression probability **(A)** *ESR1* High 7.77 vs. *ESR1* Low 10.34; **(B)** *PGR* High 7.17 vs. *PGR* Low 10.34; **(C)** *VCAN* High 7.17 vs. *VCAN* Low 10.34, and **(D)** *SFRP2* High 7.17 vs. *SFRP2* Low NA. Log-Rank test.

To investigate the link between gene expression and BCR, progression-free survival analysis (BCRFS) stratified by the gene expression level in DEGs based on BCR and CAPRA-S status was performed. We used the mean dichotomized normalized expression values for each gene to classify the expression into “Low” (below mean expression value) and “High” (above mean expression value) groups. Kaplan Meier analysis revealed a significant association between *ESR1* (Log-Rank test *P*=0.067) and *PGR* (Log-Rank test *P*=0.08), with reduced BCRFS (**Figures 5A, B**). Similarly, high expression of *VCAN* (Log-Rank test *P*=0.04) and *SFRP2* (Log-Rank test *P*=0.0006) showed a significantly shorter BCRFS (**Figures 5C, D**). Similar patterns in KM curves were identified for *INHBA*, *BBC3*, *CDKN2B*, *ORC6*, *THBS4*, and *LEFTY2*, while opposite effects were for *AR*, *LPL*, and *PIP* (**Figures S6A–I**). Similarly, progression-free survival in the validation cohorts showed comparable KM curves for *INHBA*, *BBC3*, *CDKN2B*, *ORC6*, *THBS4*, and *LEFTY2* genes (**Figures S7**, **S8**).

In the univariate Cox PH analysis, *VCAN* (HR, 2.6; 95% IC, 1.01-7.11; *P*=0.04), *SFRP2* (HR, 5.8; 95% IC, 1.87-17.97; *P*=0.002) showed a significant positive association with reduced BCRFS (**Table 4**). Both *PGR* (HR, 2.2; 95% IC, 0.88-5.84; *P*=0.08) and *ESR1* (HR, 2.3; 95% IC, 0.92-5.9; *P*=0.07) exhibited a positive correlation with reduced BCRFS that approached significance (**Table 4**). In the multivariate Cox PH analysis, *THBS4* (HR, 11.4; 95% IC, 1.1-116; *P*=0.04), *SFRP2* (HR, 27.4; 95% IC, 2.1-236.4; *P*=0.01), but not *VCAN* (HR, 1.3; 95% IC, 0.13-14.3; *P*=0.7) showed a prognostic value with reduced BCRFS when modeling the effect of each gene and clinical variables (**Table 5**).

**Table 4:** Univariate Cox PH model for BCRFS.

Variable	FMRP		TCGA		GSE54460	
	HR (95% IC)	P	HR (95% IC)	P	HR (95% IC)	P
VCAN						
VCAN high	2.6 (1.01-7.11)	0.04	1.3 (0.86-2.00)		1.5 (0.81-2.97)	
LPL						
LPL high	1.0 (0.44-2.70)		1.2 (0.82-1.88)		0.7 (0.34-1.65)	
INHBA						
INHBA high	1.5 (0.60-3.93)		1.4 (0.96-2.25)	0.07	1.58 (0.83-2.99)	
BBC3						
BBC3 high	1.9 (0.76-4.96)		1.5 (1.04-2.43)	0.03	1.79 (0.91-3.52)	
ORC6						
ORC6 high	1.6 (0.65-4.22)		2.9 (1.87-4.60)	<0.001	0.79 (0.41-1.52)	
PIP						
PIP high	1.1 (0.45-2.78)		0.9 (0.63-1.43)		1.26 (0.49-3.23)	
THBS4						
THBS4 high	2.2 (0.83-5.89)		1.1 (0.76-1.74		2.01 (1.08-3.76)	0.02
LEFTY2						
LEFTY2 high	1.4 (0.56-3.53)		1.6 (1.07-2.51)	0.02	0.90 (0.45-1.78)	
SFRP2						
SFRP2 high	5.8 (1.87-17.97)	0.002	1.1 (0.75-1.72)		1.60 (0.87-2.95)	
CDKN2B						
CDKN2B high	1.7 (0.70-4.51)		1.6 (1.09-2.54)	0.02	1.48 (0.79-2.75)	
PGR						
PGR high	2.2 (0.88-5.84)	0.08	0.5 (0.37-0.87)	<0.001	0.95 (0.52-1.73)	
ESR1						
ESR1 high	2.3 (0.92-5.99)	0.07	0.9 (0.64-1.46)		1.24 (0.65-2.35)	
AR						
AR high	0.9 (0.37-2.31)		1.1 (0.77-1.78)		1.45 (0.79-2.64)	
PSA						
6.01 to 10	1.5 (0.46-5.09)		4.8 (1.18-20.09)	0.03	0.55 (0.23-1.34)	
10.01 to 20	0.8 (0.25-2.76)		5.9 (2.16-16.46)	0.001	1.16 (0.45-3.00)	
>20	2.8 (0.32-24.94)		2.8 (0.39-20.39)		1.46 (0.56-3.80)	
pGS						
7	0.8 (0.18-3.88)		4.1 (0.56-30.76)		0.82 (0.24-2.75)	
8	3.3 (0.56-20.42)		9.7 (1.28-74.80)	0.02	1.74 (0.41-7.50)	
9	3.4 (0.48-24.77)		20.8 (2.89-151.16)	0.003	0.89 (0.19-4.14)	
10	–		19.0 (1.1-306.1)	0.03		

*Likelihood ratio test. BRFS, biochemical recurrence-free survival; CI, confidence interval; HR, hazard-ratio; pGS, Pathological Gleason Score.

**Table 5:** Multivariate Cox regression model for BCRFS.

Variable	FMRP		TCGA		GSE54460	
	HR (95% IC)	P	HR (95% IC)	P	HR (95% IC)	P
VCAN						
VCAN high	1.3 (0.13-14.30)		1.1 (0.58-2.31)		2.1 (0.44-10.63)	
LPL						
LPL high	0.2 (0.03-1.70)		0.9 (0.58-1.62)		0.5 (0.13-2.19)	
INHBA						
INHBA high	0.2 (0.03-1.33)		0.6 (0.31-1.20)		0.4 (0.07-2.64)	
BBC3						
BBC3 high	3.6 (0.53-24.54)		1.2 (0.74-1.95)		1.3 (0.53-3.60)	
ORC6						
ORC6 high	1.2 (0.21-7.63)		1.6 (0.93-2.83)		0.8 (0.30-2.26)	
PIP						
PIP high	0.2 (0.05-1.46)		0.8 (0.55-1.39)		0.3 (0.06-1.59)	
THBS4						
THBS4 high	11.4 (1.1-116.1)	0.04	0.8 (0.49-1.43)		3.6 (1.12-12.10)	0.03
LEFTY2						
LEFTY2 high	0.6 (0.09-3.82)		1.2 (0.68-2.21)		0.9 (0.27-2.96)	
SFRP2						
SFRP2 high	27.4 (2.15-349)	0.01	1.1 (0.61-1.92)		0.9 (0.22-3.56)	
CDKN2B						
CDKN2B high	0.8 (0.21-3.58)		1.6 (0.93-2.79)		1.4 (0.26-7.68)	
PGR						
PGR high	0.8 (0.13-5.41)		0.4 (0.26-0.86		1.2 (0.34-4.79)	
ESR1						
ESR1 high	1.6 (0.32-8.80)		1.5 (0.86-2.63)		1.5 (0.59-4.03)	
AR						
AR high	1.8 (0.33-10.38		1.1 (0.67-1.77)		2.2 (0.88-5.66)	
PSA						
6.01 to 10	0.6 (0.09-4.58)		2.7 (0.61-12.15)		0.3 (0.10-1.39)	
10.01 to 20	1.0 (0.12-9.73)		7.1 (2.31-21.99)	0.001	0.4 (0.08-1.95)	
>20	0.2 (0.01-7.23)		1.5 (0.20-11.68)		0.8 (0.22-3.03)	
pGS						
7	1.9 (0.26-14.72)		4.3 (0.57-32.80)		0.6 (0.11-3.67)	
8	4.5 (0.28-73.40)		7.3 (0.90-59.90)	0.06	4.8 (0.49-48.08)	
9	22.6 (0.52-990)		14.1 (1.88-106)	0.01	1.4 (0.19-11.85)	
10	–		15.7 (0.91-273)	0.05		

*Likelihood ratio test. BRFS, biochemical recurrence-free survival; CI, confidence interval; HR, hazard-ratio; pGS, Pathological Gleason Score.

## Discussion

RNA gene signature biomarker panels are well-developed in BCa (30), and this study investigated whether genes and/or pathway signatures might be common to progression in both tumor types. In this work, we utilized the NanoString BC360 curated panel to identify tumor-agnostic biological characteristics in PCa and provide prognostic information on pathways mediating progression and treatment response. When we compared patients with BCR or classified based on CAPRA-S, we identified a subset of genes related to tumor progression and TME activation.

Breast cancer treatments are more diverse than PCa, with a range of target therapies and hormone-based treatments, reflecting the heterogeneity of the various breast cancer subtypes. In contrast, radiation and surgery remain the primary options for PCa with localized disease. While there are mutations like BRCA2 in BCa that direct therapies, genetic factors play a smaller role in PCa compared to breast cancer. Since some of the various signatures derived the BC360 panel have been trained for use in BCa, it was not unexpected that there were no clear correlations between these PCas (**Figure 1A**). Across the FMRP cohort, there was a strong expression of FOXA1, an AR and ER transcription factor required to facilitate chromatin binding (72, 73). Interestingly there was strong activation of ER signaling pathways as shown by the high ER Signaling scores which capture the impact of ER pathways on downstream signaling (**Table S3**). Indeed, despite the comparatively lower expression of ESR1 to AR, the enrichment of its partner pathways and genes is coincident with higher TGFB1 expression, relevant in PCa pathobiology (74). Additionally, the bespoke signatures in the panel revealed the variability of genes related to the immune microenvironment, cell adhesion, and differentiation. The Tumor Inflammation Score (TIS) (35), an 18-gene signature that measures pre-existing but suppressed adaptive immune response in cancers for putative sensitivity to PD-1/PDL-1 blockade, showed a range of scores consistent with those across the TCGA-PRAD cohort (75). Linked to observed low expression of PD-1 and PD-L1 and other immune genes, this dataset showed these samples to be largely immune-cold and consistent with the 8% of primary PCas identified as PD-L1 positive and 32% of metastatic castrate-resistant PCa (76). The relatively immune cold phenotype of this cohort as demonstrated by the various immune-based signatures is consistent with the resulting PAM50 Luminal A classification and the notion that Luminal A BCas are generally less-immune rich than their triple negative or HER2 positive counterparts (77).

Our study classified all cases as Luminal A using the validated PAM50 gene set and algorithm (NanoString Technologies), which is inconsistent with the previous application of the molecular classifier using expression microarrays (29). In this large retrospective study (29) which included 3782 PCa cases, the distribution across the subtypes were: Luminal A (34.3%), Luminal B (28.5%), and Basal-like (32.6%). Chance and a comparatively smaller cohort, in addition to platform differences, could account for the discordance in subtype distribution shown in our study. Additionally, the study by Zhao et al. excluded the HER2 subtypes from the classification algorithm, forcing the assignment of all samples into Luminal A, Luminal B, or Basal-like categories. Collectively, these data provide caution about drawing conclusions based on one technology. Nevertheless, in a subset of cases, correlations could be found between increasing Genomic Risk Scores and less-Luminal A-like correlations. Indeed, unsupervised clustering using the PAM50 genes showed comparatively higher expression of proliferation, cell cycle, and receptor tyrosine kinase genes suggesting a more aggressive molecular phenotype associated with Luminal B, HER2-enriched and Basal-like cancers. This contrasted with the more strongly correlated Luminal A cases in this study (i.e., correlations greater than the Luminal A mean) (**Figures 1B**, **2**). To our knowledge, this is the first reported use of the PAM50 subtype raw value correlations to reveal the molecular heterogeneity within Luminal A-defined PCas. The implications of the findings revealed by the molecular subtyping as well as the Genomic Risk Scores driven by the PAM50 genes could identify those who would benefit from accelerating ADT and RT in addition to novel therapies for PCa currently being used in BCa. There are now clinical trials underway evaluating the use of CDK4/6 inhibitors in the metastatic and castration-resistant setting (reviewed by Kase et al. (78)) and the extension of the vast findings in the BCa could allow the repurposing of existing signatures for adoption in PCa. The retrospective finding of relatively higher benefit of Luminal B BCs patients to ribocilib in the MONALESSA trials (78), offers the possibility of stratifying genomically high-risk, cell-cycle driven PCa patients to CDK4/6 inhibitors. The strong role of hormone receptors in driving cellular proliferation and aberrant signaling could support the use of CDK4/6 inhibitors against a backbone of ADT, similar to current advanced BCa treatment protocols.

Indeed, as discussed above an important consideration of this study was the role of progression in the context of the expression of steroid hormone receptors (22, 25). Our analysis revealed an association between BCR status and high levels of AR expression (**Figure 1**), which was comparable to the high expression levels of *ESR1* and *PGR*, although expressed at lower levels than AR (**Figure S6**). Of particular interest was the observation that despite the overall low expression of *ESR1*, there was a strong activation of ER based on BC360 analysis (**Figure 1A**). We found that BCR patients with higher ER and PGR expression had a positive association with reduced BCRFS (**Figure 5**). Through Kaplan Meier analysis, we confirmed a positive association between *ESR1* and *PGR* with reduced BCRFS, and a positive hazard ratio was evident in Cox PH univariate analysis for *ESR1* (**Figures 5A, B**; **Table 4**). Previous studies have also speculated on the role of steroid hormone receptors (ER and PGR) in the development of PCa (77–85). In a mouse model, functional ESR1 was shown to be necessary for PCa development (86), and during the progression of PCa, *ESR1* was found at low levels in the prostatic epithelium (79) but was overexpressed in the stroma, promoting tumor progression in a paracrine manner. PGR emerged as an essential marker for estrogen-regulated growth, with some studies suggesting a positive regulatory effect of ER on PGR expression (85, 87). However, there are conflicting data on the role of PGR in PCa progression, necessitating further studies (77, 79–82). Overall, our analysis of the BC360 panel identified enrichment of the ER Signaling Signature across the samples (**Figure 1A**), suggesting activated pathways downstream of ER, even at low ER levels (**Table S3**). These findings support the idea that as PCas become androgen-insensitive, progression may be partly driven by ER-mediated pathways either in the cancer itself or through ER-mediated signaling in the stroma creating a permissive microenvironment favoring tumor progression Interrogation of the gene panel showed a prominent role of the EMT in relation to BCR. Kaplan Meier analysis indicated that high expression of *VCAN* (Log-Rank test *P*=0.04) was associated with a significantly lower BCRFS, consistent with the recognition of VCAN-mediated progression (80). *VCAN* expression is regulated by *SNAIL*, which is proposed to bind to the gene’s promoter region (88). VCAN protein expression has important roles in the tumor microenvironment and contributes to immune cell infiltration and extracellular matrix remodeling. Versican is a large proteoglycan composed of an N-terminal G1 domain, a CS attachment region, and a C terminus (or G3) selectin-like domain (82). The different components of the extracellular matrix, including the EGF receptor (EGFR), interact with the VCAN-G3 domain, which enhances cell proliferation and migration by upregulating EGFR/ERK pathway signaling. Furthermore, proteoglycans, such as VCAN, are regulated by the gene family known as ADAMTS (A Disintegrin-like And Metalloproteinase domain with Thrombospondin-1 motif), which promotes VCAN extracellular cleavage and protein activation (83). Interestingly, studies found that *VCAN* expression in PCa may be the source of cancer stem cell extracellular signaling cascade to initiate tumor formation (84). Also, a study of ADAMTS in PCa cells showed that increased TGFB1 negatively regulates ADAMTS transcripts and aids the increase of VCAN in the PCa stromal compartment (85). In our cohort, patients who experienced BCR also had significantly elevated *TGFB1* expression compared to those who did not have BCR (**Figure S5**, *p* = 0.018). Recently, seven genes, including *VCAN*, were found to be enriched in PCa extracellular matrix and were associated with BCR and bone metastasis (87). These findings draw attention to the role of the tumor microenvironment in the outcome of breast and prostate cancer. For example, earlier microarray analysis of stromal gene expression signatures in both tumor types identified a small deregulated microenvironmental gene set common to both cancers that were predictive of poor prognosis (86).

High expression of *SFRP2* (Log-Rank test *P*=0.0006) also showed an association with a significantly lower BCRFS in our retrospective cohort. *SFRP2* is part of a gene family of five glycoproteins that act as extracellular ligands of soluble Wnt ligands (89). SFRPs sequesters and inhibit frizzled (FZ) receptors and decrease WNT/β-catenin pathways activation. Several studies highlighted the role of SFRPs in stomal-to-epithelial paracrine signals and have shown that hypermethylation of this gene is associated with the inactivation of Wnt agonistic function and is frequently altered in cancer, including breast and prostate (88, 90–93). Immunohistochemical analysis of SFRP2 showed reduced expression of SFRP2 in low Gleason PCa, but diverse expression in Gleason 5 tumors (94). This study identified, among patients who experienced BCR, a group of Gleason 5 samples with strong/moderate SFRP2 expression and with a morphologic solid growth pattern. In a follow-up study (95) the authors showed a high frequency of hypermethylation of *SFRP2* and concomitant low expression of the gene in PCa (92). Earlier studies showed that *SFRP2* expression was elevated in breast tumor endothelium tissues compared to normal tissues (96). Furthermore, *SFRP2* overexpression was also associated with increased tumor size and reduced survival rates in BCa (97). Therefore, while there are discrepancies in SFRP expression likely arising because of the heterogeneity of DNA methylation in PCa, our results demonstrate a link between elevated *SFRP2* expression and cancer progression, particularly in PCa, emphasizing the role of SFRP2 in disease progression.

Finally, we identified and subsequently validated in the TGCA, the prognostic potential of ORC6, the sixth subunit of a DNA-binding complex, shown to be dysregulated in a number of neoplasms. Lin et al. (52), recently demonstrated ORC6 as prognostic for overall survival, disease-specific survival, disease-free interval, and progress-free interval in several cancers including the prostate, and that ORC6 may promote immunosuppression in a wide array of cancer.

Although this study used a well-defined workflow to underscore the potential of the NanoString BC360 panel for detecting DEGs and pathways linked to progression, our study also has specific limitations that need to be addressed in future studies. Our analysis focused on a transcriptome-derived panel tailored to specific biological processes relevant to BCa, thus not fully capturing all the pathological and molecular variables that could be unique to prostate tissue. Also, our study does not consider the influence of mutations and copy-number alterations that may modify transcriptomic signatures. Furthermore, while tissues were macrodissected to enrich for cancer cells, it is important to recognize the contributions of the surrounding tissue stroma and infiltrating immune cells to the gene expression analyses and to PCa pathogenesis. To mitigate potential statistical biases, future research will require a comprehensive analysis of a larger and more diverse PCa cohort. Our findings highlight the promise of the NanoString BC360 panel in identifying tumor-agnostic markers linked to prostate carcinogenesis and progression. They underscore the potential utility of employing tumor-agnostic biomarkers to classify patients within the realm of hormone-driven tumors such as prostate and breast cancers, thereby contributing to optimized clinical decision-making and favorable treatment results.

## Conclusion

In this study, we reasoned that PCa might involve some of the same genes and pathways of tumor progression utilized by aggressive BCas. The refinement of BCa prognostic and predictive gene expression signatures has provided crucial information for effectively using precision therapeutics in this endocrine-driven cancer (30). Tumor-agnostic biomarkers have gained interest in prognosis and treatment response studies (18). Several characteristics, such as expression signatures, DNA mismatch repair deficiency (MMR-D), and *BRCA1* status, are currently used agnostically regardless of cancer origin (22, 98, 99). Breast cancer and PCa are both proposed to be biologically related (100, 101), and both tumors share common biological features during hormone-dependent development (23, 29). Multi-omics studies in breast and prostate cancer identified common epigenome alterations in CpG islands in predicted therapeutic targets. Transcriptome-wide analyses also uncovered risk-associated miRNAs associated with hormone-dependent cancers. Thus, studying DEGs common to breast and prostate cancer may identify shared biological pathways and more robust tumor-agnostic biomarkers.

In this work, we investigated the power of the NanoString BC360 panel in identifying tumor-agnostic biological characteristics between breast and prostate cancer. We identified DEGs such as *VCAN*, *SFRP2* involved in angiogenesis and epithelial-mesenchymal transition, which can be developed as novel tumor-agnostic biomarkers for tumor progression. In further analysis, *PGR*, *ESR1*, and *VCAN* gene expression predicted relapse events. Taken together our results may support means for effectively detecting cancers that progress contribute to improved patient prognostic stratification.

## Data availability statement

The datasets presented in this study can be found in online repositories. The names of the repository/repositories and accession number(s) can be found in the article/**Supplementary Material**.

## Ethics statement

The studies involving humans were approved by Ethics Committee in Research of Hospital of Ribeirão Preto, São Paulo, Brazil (HCRP) number CAAE 60032122.8.0000.5440 and the Ethics Board of the University of Toronto (Protocol: 00043323). The studies were conducted in accordance with the local legislation and institutional requirements. The participants provided their written informed consent to participate in this study.

## Author contributions

WL: Conceptualization, Data curation, Formal Analysis, Investigation, Methodology, Project administration, Validation, Visualization, Writing – original draft, Writing – review & editing. CM: Data curation, Investigation, Methodology, Validation, Writing – review & editing. LC: Data curation, Investigation, Methodology, Writing – review & editing. FS: Data curation, Writing – review & editing. CC: Data curation, Investigation, Methodology, Validation, Writing – review & editing. AS: Data curation, Investigation, Methodology, Validation, Writing – review & editing. EW: Data curation, Investigation, Methodology, Validation, Writing – original draft. FS: Data curation, Investigation, Methodology, Validation, Writing – review & editing. FA: Data curation, Methodology, Writing – review & editing. Rd: Data curation, Investigation, Methodology, Project administration, Writing – review & editing. JS: Conceptualization, Data curation, Formal Analysis, Funding acquisition, Investigation, Methodology, Project administration, Resources, Supervision, Validation, Visualization, Writing – original draft, Writing – review & editing. JB: Conceptualization, Data curation, Formal Analysis, Funding acquisition, Investigation, Methodology, Project administration, Resources, Supervision, Validation, Visualization, Writing – original draft, Writing – review & editing.
