# Supplementary material for: Identification of tumor-agnostic biomarkers for predicting prostate cancer progression and biochemical recurrence
Source: Front Oncol. 2023 Oct 26;13:1280943. doi: 10.3389/fonc.2023.1280943 (PMC10641020; doi:10.3389/fonc.2023.1280943)
Supplement: Supplementary file 1 [file DataSheet_1.pdf]

frontiers

Supplementary Material

a

b

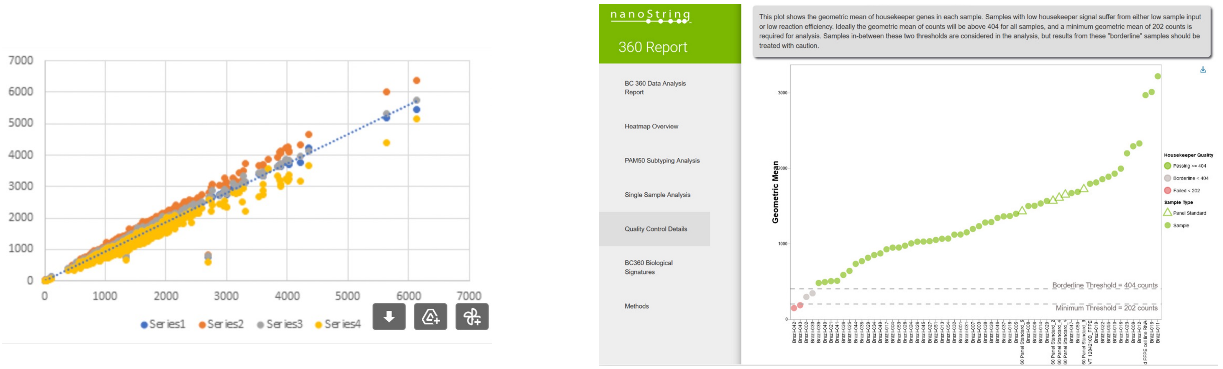

Supplementary Figure 1. QC analysis. (A) Correlation analysis between replicates for all the BC360 controls shows a good correlation between replicates and low technical variability. **(B)** RNA from all 54 samples included in the FMRP cohort were isolated and sequenced using the NanoString BC360 panel. nSolver's data quality assessment requires a threshold of 202 counts for housekeeping genes in each sample for analysis. Therefore, we removed samples 42 and 43, and only samples above the minimum threshold were used for downstream analysis (n = 51).

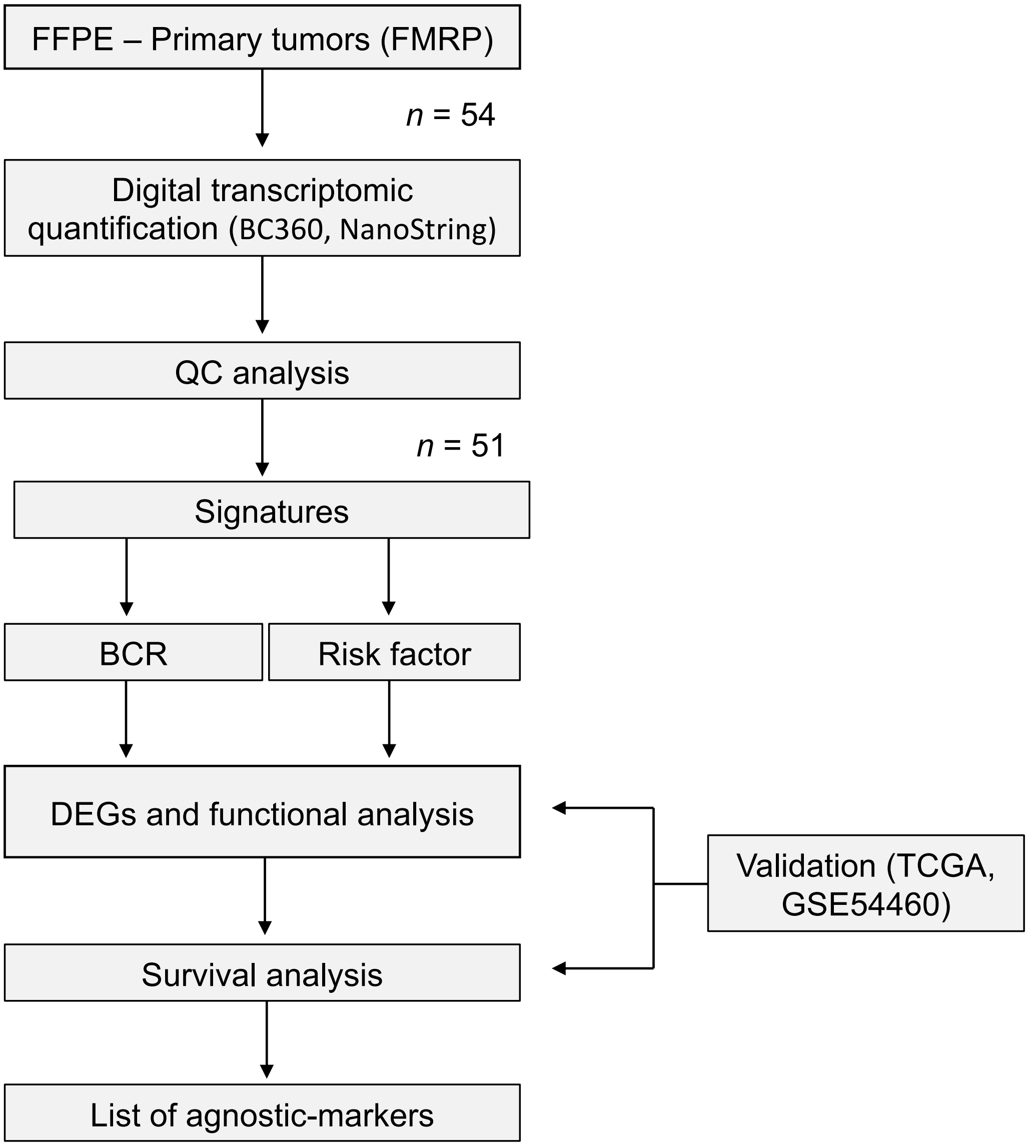

Supplementary Figure 2. Summary workflow. We conducted digital transcriptomic with BC360 NanoString panel in Formalin-Fixed Paraffin-Embedded (FFPE) tissues from Faculty of Medicine of Ribeirao Preto (FMRP) cohort (*n*=53). Two samples were removed from downstream analysis because failure quality control (QC) check. Signatures and differential analysis were perfomed in using our in-house pipeline (see methods).

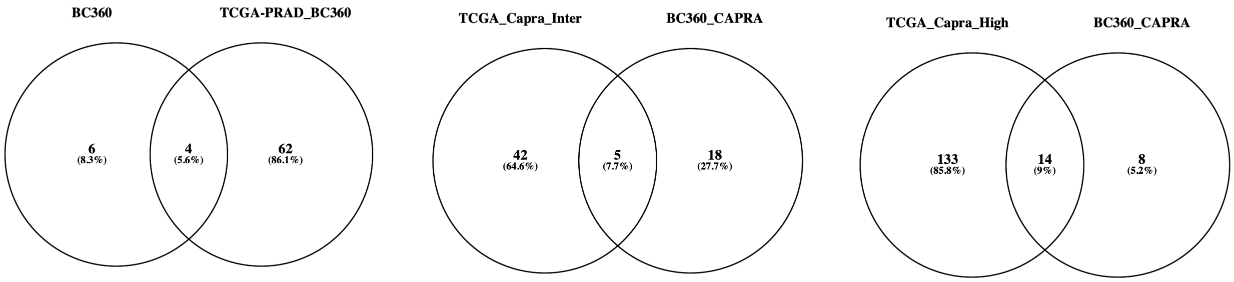

ORC6, PIP, LEFTY2, **INHBA**

ASPN, **INHBA,** KRT14, PIP

MMP11, ASPN, **INHBA,** OLFML2B, FAP, MSR1, THY1, NOTCH3, RORB, GAS1, JAG1, KRT14, CXCL10, KRT5

Supplementary Figure 3. Venn's Diagram shows the intersection of the DEGs from the NanoString BC360 panel analysis comparing FMRP and TCGA-PRAD cohorts. The genes present in all panels were highlighted. Log2FC > 0.5, P-adjusted < 0.05 (FDR).

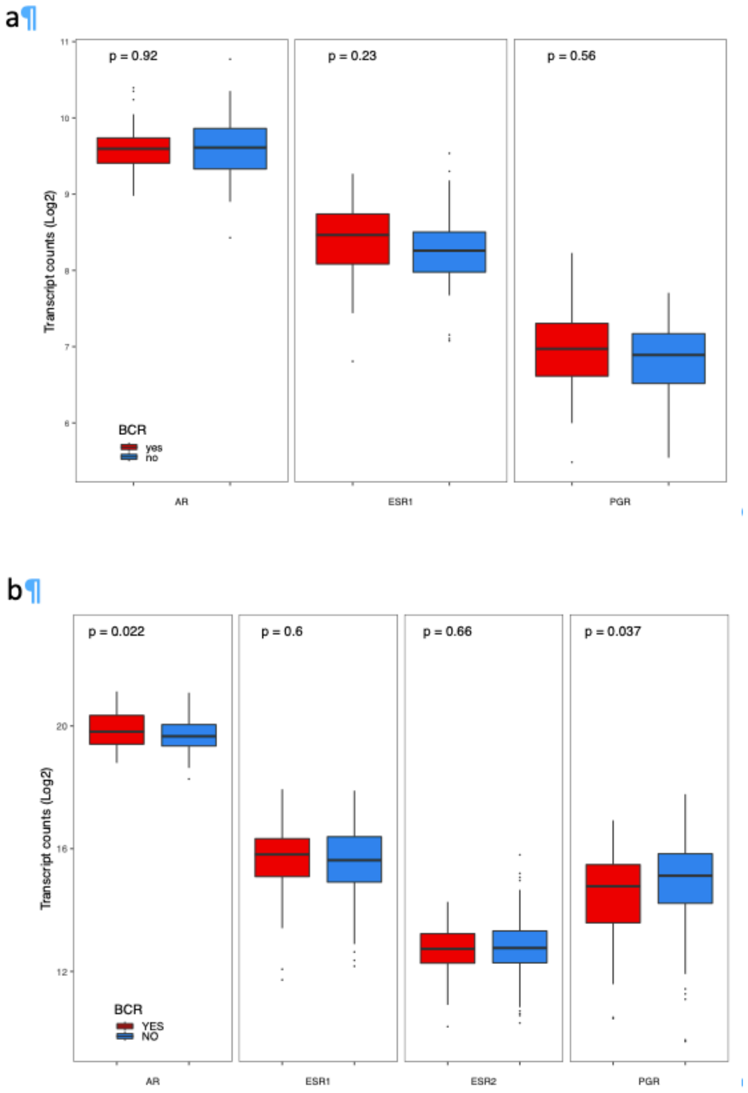

Supplementary Figure 4. Expression of hormonal receptors in prostate cancer – Box plot of the normalized log2 transformed gene counts for the androgen receptor (*AR*), estrogen receptor 1 (*ESR1*), estrogen receptor 2 (*ESR2*), and progesterone receptor (*PGR*). a) shows the expression pattern for the markers in the BC360 cohort, while b) shows the results for the TCGA-PRAD cohort. The analysis used no biochemical recurrence as a control. (Mann–Whitney U test, *, $p < 0.05$).

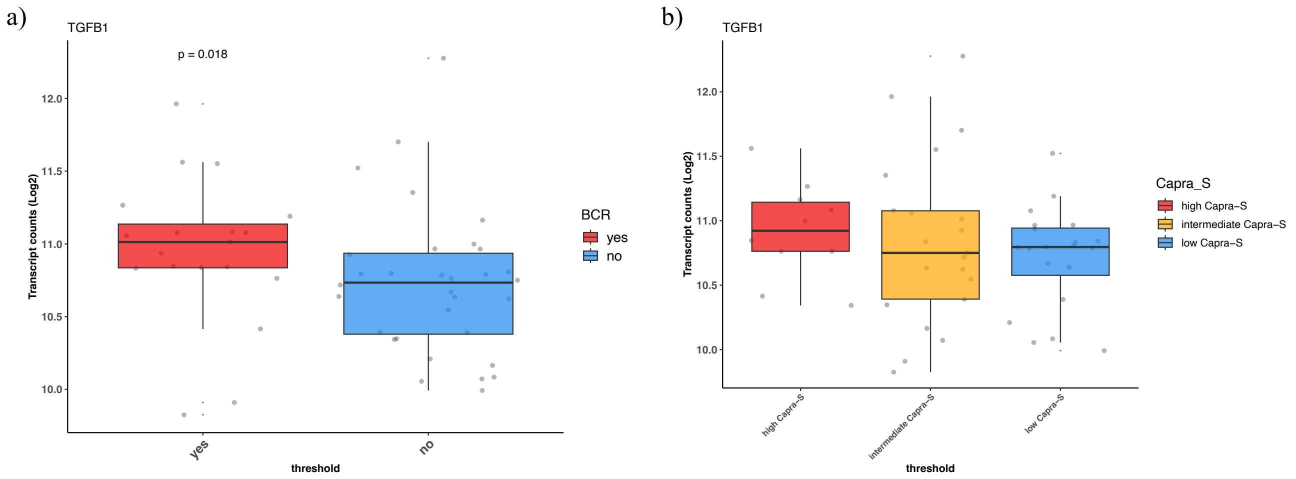

Supplementary Figure 5. Expression of *TGFB1* in prostate cancer – Box plot of the normalized log2 transformed gene counts for the Transforming Growth Factor Beta 1 (*TGFβ1*). a) shows the expression pattern for the BCR, while b) shows the results for the CAPRA-S score in the FMRP cohort. The normalized log2 transformed transcript counts show a significant increase in *TGFβ1* gene counts for BCR patients (p = 0.018). The analysis used no biochemical recurrence as a control. (Mann–Whitney U test).

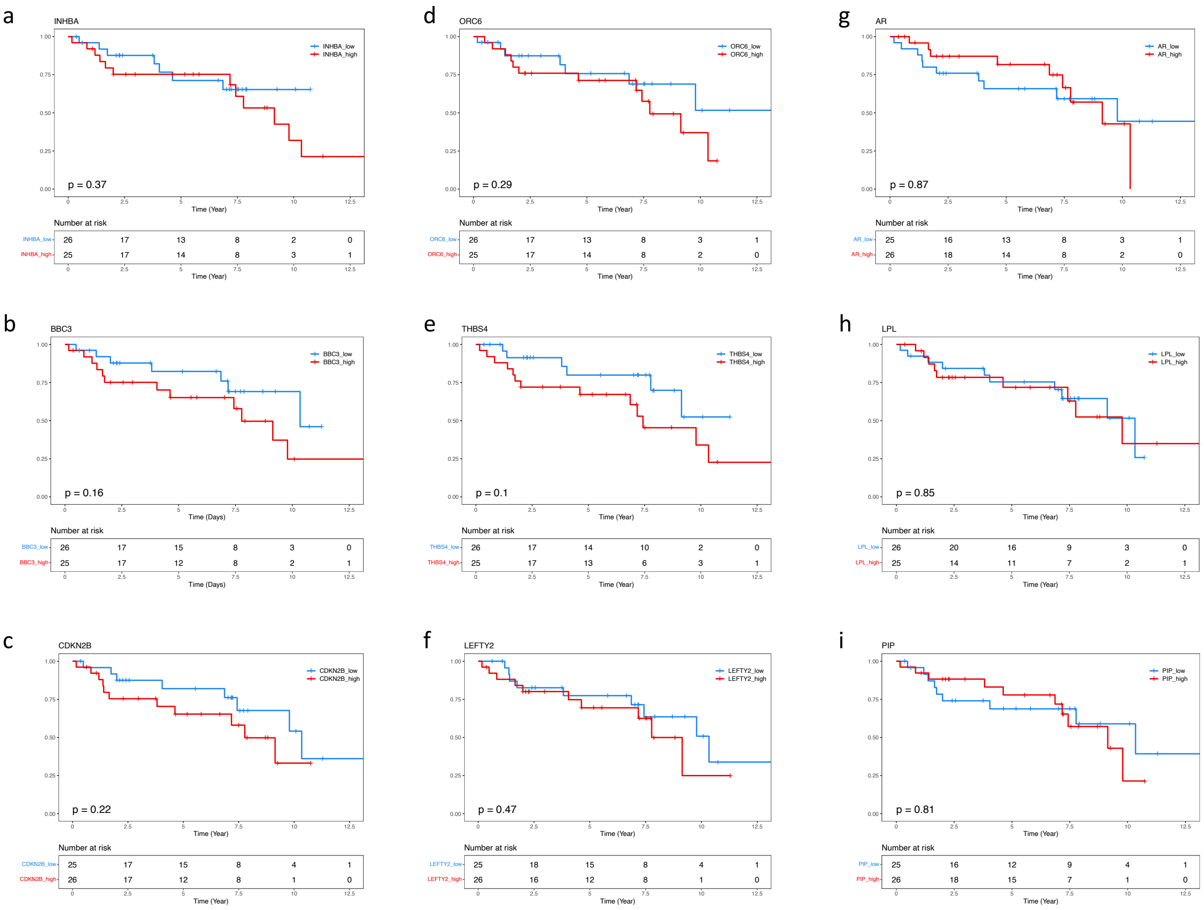

Supplementary Figure 6. Kaplen-Maier curves illustrate biochemical recurrence free-survival for *INHBA, BBC3, CDKN2B, ORC6, THBS4, LEFTY2, AR, LPL,* and *PIP* in the FMRP cohort. Results using the progression-free survival interval for BCR. Median 50% progression probability **(a)** *INHBA* High 9.14 vs. *INHBA* Low NA, **(b)** *BBC3* High 7.77 vs. *BBC3* Low 10.34, **(c)** *CDKN2B* High 7.77 vs. *CDKN2B* Low 10.34, **(d)** *ORC6* High 7.77 vs. *ORC6* Low NA, **(e)** *THBS4* High 7.43 vs. *THBS4* Low NA, **(f)** *LEFTY2* High 8.46 vs. *LEFTY2* Low 10.34, **(g)** *AR* High 9.14 vs. *AR* Low 9.79, **(h)** *LPL* High 9.79 vs. *LPL* Low 10.34, **(i)** *PIP* High 9.14 vs. *PIP* Low 10.34. Log-Rank Test.

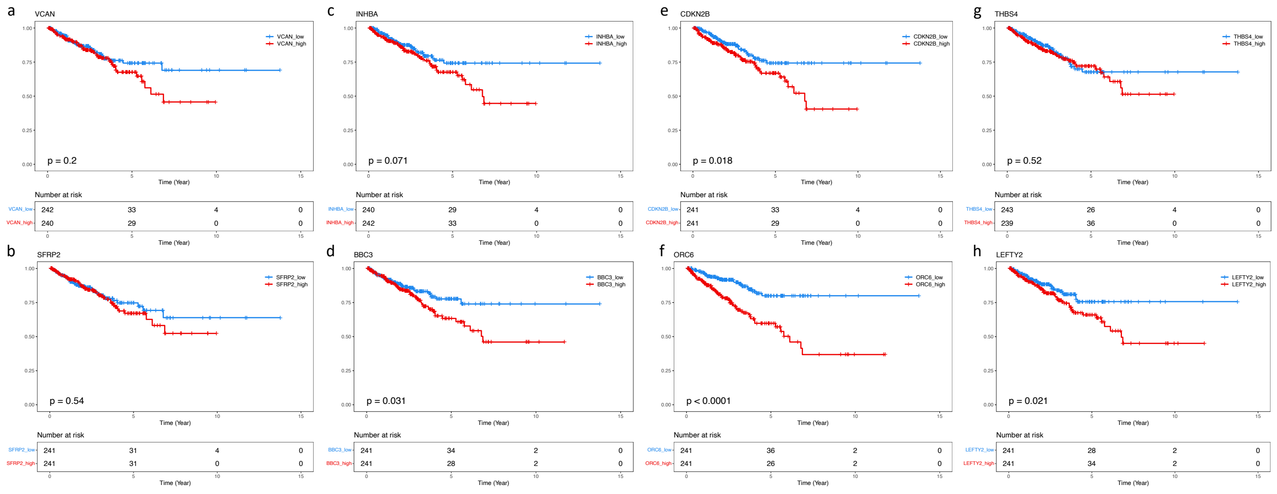

Supplementary Figure 7. Kapler-Maier curves illustrate biochemical recurrence free-survival for *VCAN*, *SFRP2*, *INHBA*, *BBC3*, *CDKN2B*, *ORC6*, *THBS4*, and *LEFTY2* in the TCGA-PRAD cohort. Results using the progression-free interval (PFI) for BCR from TCGA. Median 50% progression probability **(a)** *VCAN* High 6.86 vs. *VCAN* Low NA, **(b)** *SFRP2* High NA vs. *SFRP2* Low NA, **(c)** *INHBA* High 6.78 vs. *INHBA* Low NA, **(d)** *BBC3* High 6.86 vs. *BBC3* Low NA, **(e)** *CDKN2B* High 6.78 vs. *CDKN2B* Low NA, **(f)** *ORC6* High 6.12 vs. *ORC6* Low NA, **(g)** *THBS4* High NA vs. *THBS4* Low NA, **(h)** *LEFTY2* High 6.78 vs. *LEFTY2* Low NA. Log-Rank test.

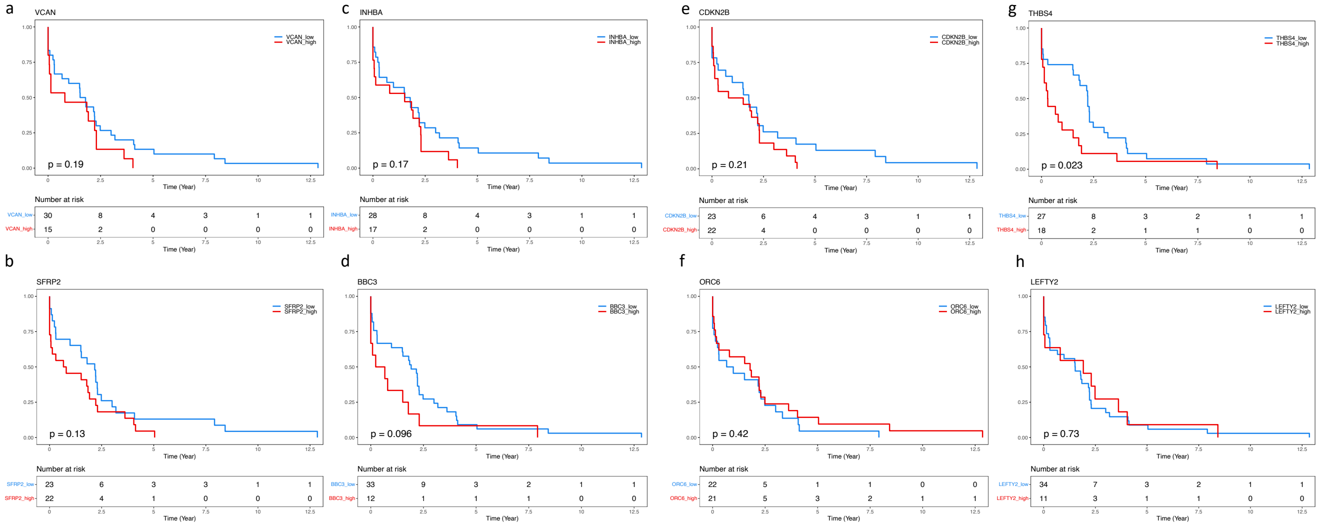

Supplementary Figure 8. Kapler-Maier curves illustrate biochemical recurrence free-survival for *VCAN*, *SFRP2*, *INHBA*, *BBC3*, *CDKN2B*, *ORC6*, *THBS4*, and *LEFTY2* in the GSE54460 cohort. Results using the progression-free interval (PFI) for BCR from TCGA. Median 50% progression probability **(a)** *VCAN* High 1.65 vs. *VCAN* Low 0.8, **(b)** *SFRP2* High 0.7 vs. *SFRP2* Low 2.1, **(c)** *INHBA* High 1.52 vs. *INHBA* Low 1.65, **(d)** *BBC3* High 0.45 vs. *BBC3* Low 1.92, **(e)** *CDKN2B* High 1.16 vs. *CDKN2B* Low 1.78, **(f)** *ORC6* High 1.53 vs. *ORC6* Low 1.65, **(g)** *THBS4* High 0.29 vs. *THBS4* Low 2.2, **(h)** *LEFTY2* High 1.92 vs. *LEFTY2* Low 1.52. Log-Rank test.
