# Supplementary material for: Identification of tumor-agnostic biomarkers for predicting prostate cancer progression and biochemical recurrence
Source: Front Oncol. 2023 Oct 26;13:1280943. doi: 10.3389/fonc.2023.1280943 (PMC10641020; doi:10.3389/fonc.2023.1280943)
Supplement: Supplementary file 2 [file Table_1.docx]

Supplementary Material

|  | **FMRP** | **TCGA** | **GSE54460** |
| --- | --- | --- | --- |
| **Variable** | **n** | **n** | **N** |
| **BCR** |  |  |  |
| no | 32 | 363 | 51 |
| yes | 19 | 57 | 49 |
| NA | - | 68 | 6 |
| **Capra-S** |  |  |  |
| low Capra-S | 20 | 184 | 66 |
| Intermediate Capra-S | 21 | 196 | 29 |
| High Capra-S | 10 | 108 | 5 |
| **pGS** |  |  |  |
| 5 | - | - | 1 |
| 6 | 8 | 44 | 10 |
| 7 | 37 | 242 | 75 |
| 8 | 4 | 62 | 10 |
| 9 | 2 | 136 | 4 |
| 10 | - | 3 | - |
| NA | - | 1 | 6 |
| **ISUP** |  |  |  |
| 1 | 8 | - |  |
| 2 | 24 | - |  |
| 3 | 13 | - |  |
| 4 | 2 | - |  |
| 5 | 4 | - |  |
| **TNM** |  |  |  |
| pT1c | - | - | 12 |
| pT2 | 2 | - | 10 |
| pT2a | 2 | 13 | 21 |
| pT2b | 3 | 10 | - |
| pT2c | 30 | 162 | 30 |
| pT3a | 6 | 155 | - |
| pT3b | 7 | 131 | 9 |
| T4 | - | 11 | - |
| NA | - | 6 | 24 |
| **pre-surgical PSA(ng/ml)** |  |  |  |
| 0 to 6 | 16 | 382 | 32 |
| 6.01 to 10 | 12 | 6 | 35 |
| 10.01 to 20 | 19 | 11 | 19 |
| >20 | 3 | 4 | 11 |
| NA | 1 | 85 | 9 |
| **Surgical Margin** |  |  |  |
| 0 | 37 | 309 | - |
| 1 | 14 | 150 | - |
| **Extraprostatic Invasion** |  |  |  |
| 0 | 39 | 185 | - |
| 1 | 12 | 297 | - |
| NA | - | 6 | - |
| **Vesicular Invasion** |  |  |  |
| 0 | 50 | 58 | - |
| 1 | 1 | 19 | - |
| NA | - | 411 | - |
| **Average** |  |  |  |
| **Age at surgery** |  |  |  |
| 66 |  | 61 | 61.1 |

**Supplementary Table 1. Prostate cancer tissue samples**. BCR, biochemical recurrence; CAPRA-S, Cancer of the Prostate Risk Assessment Score; pGS, pathologic Gleason Score; ISUP, International Society of Urological Pathology Score; TNM, Classification of Malignant Tumors.
