# Supplementary material for: Identification of tumor-agnostic biomarkers for predicting prostate cancer progression and biochemical recurrence
Source: Front Oncol. 2023 Oct 26;13:1280943. doi: 10.3389/fonc.2023.1280943 (PMC10641020; doi:10.3389/fonc.2023.1280943)
Supplement: Supplementary file 3 [file Table_2.docx]

Supplementary Material

| **Sample ID** | **% tumor** | **comments** |
| --- | --- | --- |
| 1 | 90 | One medium area with stromal intermingled |
| 2 | 85 | One big area with with stromal intermingled |
| 3 | 95 | One big area with immune infiltration |
| 4 | 70 | One small area with stromal intermingled |
| 5 | 70 and 85 | Two different areas, and with stromal intermingled |
| 6 | 80 | One big area with with stromal intermingled |
| 7 | 95 | Four areas with low stromal intermingled |
| 8 | 85 | Stroma on the periphery of the selected area |
| 9 | 95 | One big area with low stromal intermingled |
| 10 | 98 | One medium area with low stromal intermingled |
| 11 | 98 | One small area with low stromal intermingled |
| 12 | 95 | Three areas with low stromal intermingled |
| 13 | 85 | One medium area with stromal intermingled |
| 14 | 90 | One medium area with stromal intermingled |
| 15 | 98 | One small area with low stromal intermingled |
| 16 | 98 | One small area with low stromal intermingled |
| 17 | 95 | One small area with low stromal intermingled |
| 18 | 85 | Stroma on the periphery of the selected area |
| 19 | 95 | One small area |
| 20 | 95 | Two big areas with low stromal intermingled |
| 21 | 90 | One big area with stromal intermingled and immune infiltration |
| 22 | 90 | One medium area with stromal intermingled and immune infiltration |
| 23 | 90 | One big area with stromal intermingled and immune infiltration |
| 24 | 95 | One medium area with low stromal intermingled |
| 25 | 95 | One medium area with low stromal intermingled |
| 26 | 85 | One medium area with stromal intermingled |
| 27 | 95 and 85 | Two different areas, and with stromal intermingled |
| 28 | 70 | One small area and with stromal intermingled |
| 29 | 90 | One small area with stromal intermingled and immune infiltration |
| 30 | 90 | One small area with stromal intermingled |
| 31 | 90 | Two areas with stromal intermingled |
| 32 | 95 | One small area with low stromal intermingled |
| 33 | 98 | One small area with low stromal intermingled |
| 34 | 90 | One medium area with stromal intermingled |
| 35 | 95 | Two small areas with low stromal intermingled |
| 36 | 95 | Two small areas with low stromal intermingled |
| 37 | 85 | One small area with stromal intermingled |
| 38 | 95 | One small area with low stromal intermingled |
| 39 | 95 | One small area with low stromal intermingled |
| 40 | 95 | Two small areas with low stromal intermingled |
| 41 | 90 | One big area with stromal intermingled |
| 44 | 95 | One medium area with low stromal intermingled and immune infiltration |
| 45 | 95 | Three small areas with low stromal intermingled |
| 46 | 95 | One medium area with low stromal intermingled and immune infiltration |
| 47 | 95 | Three medium areas with low stromal intermingled |
| 49 | 70 and 90 | Two different areas, and with stromal intermingled |
| 50 | 95 | One medium area with low stromal intermingled |
| 51 | 90 | Two small areas with stromal intermingled |
| 53 | 98 | One medium area with low stromal intermingled and immune infiltration |
| 54 | 95 | One medium area with low stromal intermingled |
| 55 | 95 | One medium area with low stromal intermingled |

**Supplementary Table 2. Tumor purity.** Percentage of tumor cells in each within the marked tumor area.
