# Supplementary material for: Identification of tumor-agnostic biomarkers for predicting prostate cancer progression and biochemical recurrence
Source: Front Oncol. 2023 Oct 26;13:1280943. doi: 10.3389/fonc.2023.1280943 (PMC10641020; doi:10.3389/fonc.2023.1280943)
Supplement: Supplementary file 5 [file Table_4.docx]

Supplementary Material

| Sample ID | Subtype | Basal Correlation | LumA Correlation | LumB Correlation | HER2-E Correlation | Genomic Risk |
| --- | --- | --- | --- | --- | --- | --- |
| Pooled FFPE cell line RNA | Basal | 0.63 | -0.597 | -0.433 | 0.196 | 58 |
| BioIVT 1284210B_FFPE | LumA | -0.568 | 0.652 | -0.148 | -0.421 | 5 |
| Brazil-055 | LumA | -0.564 | 0.705 | -0.178 | -0.4 | 0 |
| Brazil-054 | LumA | -0.489 | 0.655 | -0.225 | -0.395 | 0 |
| Brazil-053 | LumA | -0.461 | 0.62 | -0.185 | -0.276 | 6 |
| Brazil-051 | LumA | -0.508 | 0.714 | -0.201 | -0.363 | 0 |
| Brazil-050 | LumA | -0.451 | 0.626 | -0.201 | -0.493 | 4 |
| Brazil-049 | LumA | -0.446 | 0.691 | -0.263 | -0.386 | 0 |
| Brazil-047 | LumA | -0.578 | 0.606 | -0.023 | -0.338 | 8 |
| Brazil-046 | LumA | -0.536 | 0.696 | -0.132 | -0.325 | 5 |
| Brazil-045 | LumA | -0.543 | 0.739 | -0.187 | -0.387 | 2 |
| Brazil-044 | LumA | -0.459 | 0.596 | -0.199 | -0.335 | 8 |
| Brazil-041 | LumA | -0.49 | 0.645 | -0.149 | -0.326 | 8 |
| Brazil-040 | LumA | -0.461 | 0.66 | -0.228 | -0.334 | 6 |
| Brazil-039 | LumA | -0.407 | 0.646 | -0.286 | -0.355 | 1 |
| Brazil-038 | LumA | -0.564 | 0.708 | -0.159 | -0.418 | 1 |
| Brazil-037 | LumA | -0.517 | 0.709 | -0.172 | -0.361 | 1 |
| Brazil-036 | LumA | -0.483 | 0.675 | -0.208 | -0.334 | 3 |
| Brazil-035 | LumA | -0.469 | 0.674 | -0.177 | -0.32 | 6 |
| Brazil-034 | LumA | -0.455 | 0.652 | -0.26 | -0.297 | 0 |
| Brazil-033 | LumA | -0.385 | 0.634 | -0.253 | -0.364 | 6 |
| Brazil-032 | LumA | -0.51 | 0.683 | -0.212 | -0.407 | 0 |
| Brazil-031 | LumA | -0.513 | 0.74 | -0.239 | -0.41 | 0 |
| Brazil-030 | LumA | -0.519 | 0.674 | -0.23 | -0.439 | 0 |
| Brazil-029 | LumA | -0.481 | 0.703 | -0.216 | -0.38 | 2 |
| Brazil-028 | LumA | -0.524 | 0.735 | -0.207 | -0.415 | 0 |
| Brazil-027 | LumA | -0.515 | 0.687 | -0.175 | -0.371 | 3 |
| Brazil-026 | LumA | -0.453 | 0.686 | -0.267 | -0.378 | 0 |
| Brazil-025 | LumA | -0.499 | 0.636 | -0.137 | -0.337 | 7 |
| Brazil-024 | LumA | -0.481 | 0.701 | -0.237 | -0.398 | 0 |
| Brazil-023 | LumA | -0.557 | 0.685 | -0.18 | -0.424 | 0 |
| Brazil-022 | LumA | -0.471 | 0.625 | -0.234 | -0.374 | 4 |
| Brazil-021 | LumA | -0.424 | 0.676 | -0.232 | -0.34 | 3 |
| Brazil-020 | LumA | -0.389 | 0.514 | -0.119 | -0.242 | 18 |
| Brazil-019 | LumA | -0.403 | 0.339 | 0.106 | -0.11 | 26 |
| Brazil-018 | LumA | -0.488 | 0.7 | -0.242 | -0.408 | 0 |
| Brazil-017 | LumA | -0.527 | 0.698 | -0.178 | -0.414 | 0 |
| Brazil-016 | LumA | -0.509 | 0.696 | -0.227 | -0.333 | 0 |
| Brazil-015 | LumA | -0.538 | 0.715 | -0.198 | -0.415 | 0 |
| Brazil-014 | LumA | -0.566 | 0.691 | -0.142 | -0.301 | 4 |
| Brazil-013 | LumA | -0.509 | 0.711 | -0.216 | -0.378 | 0 |
| Brazil-012 | LumA | -0.609 | 0.715 | -0.13 | -0.421 | 0 |
| Brazil-011 | LumA | -0.497 | 0.453 | -0.036 | -0.182 | 22 |
| Brazil-010 | LumA | -0.47 | 0.637 | -0.224 | -0.304 | 2 |
| Brazil-009 | LumA | -0.631 | 0.71 | -0.101 | -0.352 | 1 |
| Brazil-008 | LumA | -0.591 | 0.679 | -0.071 | -0.416 | 5 |
| Brazil-007 | LumA | -0.411 | 0.659 | -0.217 | -0.328 | 8 |
| Brazil-006 | LumA | -0.498 | 0.67 | -0.207 | -0.375 | 6 |
| Brazil-005 | LumA | -0.525 | 0.67 | -0.148 | -0.342 | 6 |
| Brazil-004 | LumA | -0.443 | 0.702 | -0.286 | -0.402 | 0 |
| Brazil-003 | LumA | -0.163 | 0.206 | -0.221 | -0.238 | 25 |
| Brazil-002 | LumA | -0.459 | 0.646 | -0.191 | -0.326 | 7 |
| Brazil-001 | LumA | -0.471 | 0.677 | -0.258 | -0.413 | 0 |

**Supplementary table 4. PAM50 classifier for BC360.** Values showed for each subtype for all PCa samples and two control samples (Pooled FFPE cell line RNA and BioIVT 1284210B_FFPE). In our work, the PAM50 classifier identified all 53 cases as luminal A, but in four patients with the shift for more LumB (Brazil-003, -011, -019, and -020).
