# Supplementary material for: Identification of tumor-agnostic biomarkers for predicting prostate cancer progression and biochemical recurrence
Source: Front Oncol. 2023 Oct 26;13:1280943. doi: 10.3389/fonc.2023.1280943 (PMC10641020; doi:10.3389/fonc.2023.1280943)
Supplement: Supplementary file 6 [file Table_5.docx]

Supplementary Material

|  | **Term** | **Adjusted P-value** | **Genes** |
| --- | --- | --- | --- |
| **BCR** | G2-M Checkpoint | 3.19E-23 | *TOP2A; POLQ; UBE2C; KIF23; TTK; MKI67; AURKB; AURKA; CCNA2; CENPF; ORC6; PTTG1; ESPL1; EXO1; PRC1; E2F1; CDK1; BIRC5; MYBL2; KIF2C* |
|  | E2F Targets | 4.65E-17 | *TOP2A; CDKN1A; MKI67; AURKB; AURKA; MELK; ORC6; PTTG1; ESPL1; CDK1; BIRC5; MYBL2; KIF2C; TRIP13; DLGAP5; SPC25* |
|  | Mitotic Spindle | 2.14E-11 | *TOP2A; ANLN; CENPF; ESPL1; PRC1; CDK1; BIRC5; KIF23; KIF2C; TTK; DLGAP5; AURKA* |
|  | Epithelial Mesenchymal Transition | 3.96E-4 | *GREM1; SFRP4; CXCL8; GADD45B; INHBA; THBS2* |
|  | TNF-alpha Signaling via NF-kB | 0.002 | *CDKN1A; GADD45B; CCL2; INHBA; PTGS2* |
|  | Inflammatory Response | 0.002 | *CDKN1A; CXCL8; CCL2; INHBA; SELE* |
|  | Spermatogenesis | 0.004 | *CDK1; KIF2C; TTK; AURKA* |
|  | KRAS Signaling Dn | 0.016 | *TLX1; HNF1A; KRT5; CACNG1* |
|  | PI3K/AKT/mTOR Signaling | 0.017 | *CDKN1A; E2F1; CDK1* |

**Supplementary table 5. Enrichment analysis of DEGs from TCGA cohort panel.** List of ORA enriched analysis using MSigDB pathways terms from DEGs associated with BCR. The analysis used no biochemical recurrence as a control.
