# Supplementary material for: Identification of tumor-agnostic biomarkers for predicting prostate cancer progression and biochemical recurrence
Source: Front Oncol. 2023 Oct 26;13:1280943. doi: 10.3389/fonc.2023.1280943 (PMC10641020; doi:10.3389/fonc.2023.1280943)
Supplement: Supplementary file 7 [file Table_6.docx]

Supplementary Material

|  | **Term** | **Adjusted P-value** | **Genes** |
| --- | --- | --- | --- |
| **CAPRA_S Intermediate** | G2-M Checkpoint | 4.03E-16 | *TOP2A; POLQ; UBE2C; MKI67; AURKA; CCNA2; CENPF; ORC6; ESPL1; EXO1; CDK1; BIRC5; MYBL2; CDKN3* |
|  | E2F Targets | 3.44E-13 | *TOP2A; ORC6; RRM2; MELK; ESPL1; CDK1; BIRC5; MYBL2; MKI67; DLGAP5; AURKA; CDKN3* |
|  | Mitotic Spindle | 1.56E-7 | *TOP2A; ANLN; CENPF; ESPL1; CDK1; BIRC5; DLGAP5; AURKA* |
|  | Epithelial Mesenchymal Transition | 2.52E-6 | *COMP; SFRP4; COL11A1; WNT5A; MMP3; INHBA; THBS2* |
|  | KRAS Signaling Up | 0.006 | *MMP11; RELN; INHBA; ETV4* |
|  | Spermatogenesis | 0.014 | *CDK1; AURKA; CDKN3* |
|  | Coagulation | 0.014 | *COMP; MMP11; MMP3* |
| **CAPRA_S High** | G2-M Checkpoint | 6.43E-25 | *TOP2A; TTK; KIF11; MKI67; AURKB; AURKA; CDC20; ORC6; PTTG1; EXO1; E2F1; RAD54L; MYBL2; POLQ; UBE2C; EGF; KIF23; CDC6; CCNA2; CENPF; ESPL1; CDK4; PRC1; CDK1; BIRC5; KIF2C; CDKN3* |
|  | E2F Targets | 1.95E-18 | *TOP2A; RRM2; CDCA8; MKI67; AURKB; AURKA; CDC20; MELK; ORC6; PTTG1; ESPL1; CDK4; UBE2T; CDK1; DEPDC1; BIRC5; MYBL2; KIF2C; TRIP13; DLGAP5; CDKN3; SPC25* |
|  | Epithelial Mesenchymal Transition | 4.03E-9 | *COL11A1; WNT5A; MMP3; THY1; INHBA; THBS2; COMP; GREM1; SFRP4; VCAN; FAP; ADAM12; SPP1; GAS1* |
|  | Mitotic Spindle | 3.15E-8 | *TOP2A; KIF23; TTK; KIF11; AURKA; ANLN; CENPF; ESPL1; PRC1; CDK1; BIRC5; KIF2C; DLGAP5* |
|  | KRAS Signaling Up | 2.52E-6 | *CXCL10; MMP11; MYCN; RELN; SPP1; PLAT; SOX9; INHBA; ETV4; EREG; GADD45G* |
|  | Inflammatory Response | 1.34E-4 | *MSR1; CXCL10; BTG2; CXCL9; TNFAIP6; CLEC5A; NDP; INHBA; EREG* |
|  | Angiogenesis | 9.03E4 | *VCAN; JAG1; SPP1; LPL* |
|  | Coagulation | 0.003 | *COMP; CFD; MMP11; MMP3; PLAT; LEFTY2* |
|  | Estrogen Response Late | 0.003 | *CDC20; SCUBE2; TOP2A; TFF3; CDC6; ANXA9; DHRS2* |
|  | Glycolysis | 0.003 | *EFNA3; VCAN; CDK1; TFF3; DEPDC1; SOX9; AURKA* |
|  | Notch Signaling | 0.006 | *NOTCH3; JAG1; WNT5A* |
|  | Spermatogenesis | 0.011 | *CDK1; KIF2C; TTK; AURKA; CDKN3* |
|  | IL-2/STAT5 Signaling | 0.011 | *CXCL10; MUC1; IL2RA; SPP1; CDC6; ETV4* |
|  | TNF-alpha Signaling via NF-kB | 0.011 | *CXCL10; BTG2; JAG1; TNFAIP6; LAMB3; INHBA* |
|  | Allograft Rejection | 0.011 | *CXCL9; HLA-DMA; IL2RA; THY1; INHBA; EREG* |
|  | Apical Surface | 0.011 | *GAS1; THY1; GATA3* |
|  | Myogenesis | 0.039 | *CFD; ADAM12; NCAM1; CACNG1; CRYAB* |
|  | Interferon Gamma Response | 0.039 | *CXCL10; CXCL9; HLA-DMA; TNFAIP6; OAS3* |
|  | KRAS Signaling Dn | 0.039 | *BTG2; EGF; KRT5; HNF1A; CACNG1* |

**Supplementary table 6. Enrichment analysis of DEGs from TCGA cohort panel.** List of ORA enriched analysis using MSigDB pathways terms from DEGs associated with CAPRA-S. The analysis used CAPRA-S “low” as a reference.
