# Supplementary material for: Identification of tumor-agnostic biomarkers for predicting prostate cancer progression and biochemical recurrence
Source: Front Oncol. 2023 Oct 26;13:1280943. doi: 10.3389/fonc.2023.1280943 (PMC10641020; doi:10.3389/fonc.2023.1280943)
Supplement: Supplementary file 8 [file Table_7.docx]

Supplementary Material

|  | **Term** | **Adjusted P-value** | **Genes** |
| --- | --- | --- | --- |
| **BCR** | Interferon Gamma Response | 3,68E-04 | *OAS3;ISG15;PTGS2* |
|  | Spermatogenesis | 0,005 | *PARP2;MTOR* |
|  | Interferon Alpha Response | 0,06 | *ISG15* |

**Supplementary table 7. Enrichment analysis of DEGs from GSE54460 cohort panel.** List of ORA enriched analysis using MSigDB pathways terms from DEGs associated with BCR. The analysis used no biochemical recurrence as a control.
